# Turbulence drives seabed modification by offshore windfarms

**DOI:** 10.1038/s41467-026-73089-x

**Published:** 2026-06-03

**Authors:** Christopher A. Unsworth, Connor J. McCarron, Richard J. S. Whitehouse, Thomas D. G. Benson, Ignacio Barranco, Michael A. Clare, James J. Waggitt, Lisa Skein, Veerle A. I. Huvenne, Martin J. Austin, Katrien J. J. Van Landeghem

**Affiliations:** 1 School of Ocean Sciences, Bangor University, Menai Bridge, Isle of Anglesey, United Kingdom; 2 Coasts and Oceans, HR Wallingford, Howbery Park, Wallingford, United Kingdom; 3 Technology Centre for Offshore and Marine, Singapore (TCOMS). 2 Prince George’s Pk, Singapore, Singapore; 4 National Oceanography Centre, Southampton, UK; 5 Institute for Coastal and Marine Research, Nelson Mandela University, Gqeberha, South Africa

**Keywords:** Physical oceanography, Wind energy, Environmental impact

## Abstract

The drive to cut carbon emissions and harness renewable energy has spurred rapid industrialization of the seas. Current estimates of seabed modification due to offshore windfarms (ca. 1% of a windfarm’s area) do not account for enhanced seabed mobility due to monopile wakes. Here we demonstrate how turbulence emanating from windfarm infrastructure mobilizes seabed sediments. Whilst mean flow conditions in our laboratory experiment could mobilize fine sand, near-bed turbulence from monopile’s wake could mobilize sand 17 monopile diameters (*D*) downstream. Related bedform formation coincided spatially with downwelling of the monopile’s turbulent wake, indicating that the 3D wake flow structure both creates and defines enhanced sediment mobility. We introduce a method for predicting this region of enhanced transport capacity by combining bed shear stress from near-bed turbulence with that from mean flow; and our field evidence at an operational offshore windfarm validates this approach. Our results show bed stress amplification is expected cover 3-8% of a typical windfarm area. Enhanced seabed mobility can alter seabed habitats and should be factored into impact assessment and marine planning.

## Abstract (web summary)

This study investigates the influence of turbine foundations on the seabed within windfarms finding that it has a larger impact than previously thought. The results show that bed stress amplification covers 3−8% of a typical windfarm area.

## Introduction

Driven by the desire to meet global climate goals, the world’s offshore wind power capacity will continue to increase globally, with plans to install 380 GW of offshore wind capacity by 2030 and 2000 GW by 2050^1^. The planned acceleration of offshore wind currently faces challenges, one of which is project delays due to uncertainty in the evidence of effective and strategic compensation measures. Accelerated permitting protocols have been widely recognized as one of the fastest ways to unlock offshore wind potential and reach the targets set during COP-26 and COP-27^2^, but guidance is needed to render impact assessments more appropriate to minimize risk to marine life and to support nature-positive approaches to infrastructure installation^3,4^. To support effective decision-making across managerial, governmental, political, and legal components, a strategic goal is needed to include improvements to marine ecosystems, underpinned by well-accepted state-of-the-art scientific research. This drives the need to understand the potential unintended consequences of the industrialization of the seas, and to help identify opportunities for nature recovery. In doing so, we can help underpin a flexible library of options for mitigation and compensation in ways that safeguard ecological integrity and function.

Global shelf seas support most existing offshore windfarms. For example, the Northwest European shelf currently hosts circa 6000 offshore windfarm monopiles, an increase of 2000 since 2017. The Chinese coastline hosts the largest proportion of global offshore wind power^1^, and new regions along the Australian, Brazilian, SE Asian and Eastern USA coasts are being considered for development^1,5^. There is a growing body of research into the effects of current and future developments^6–14^. However, one particularly overlooked research area is the potential for change to the seabed itself.

For reasons of efficiency and cost-effectiveness, monopile foundations are often the preferred (4 in 5) foundation design for depths <60 m^5,15,16^. Monopiles founded in the seabed divert the flow of water and cause increased bed shear stress, raising concern for monopile scour immediately adjacent to the monopile^17^. The scour around fixed-bed offshore windfarms is extensively studied^18–21^, primarily for foundation stability, leading to scour mitigation practices like rock armoring^22–25^. However, less well researched are the potential effects of the wake recovery flow downstream of a monopile, which is a complex three-dimensional flow structure^19,23,26,27^ that can alter bed shear stress^28,29^.

Existing quantifications of the recovery distance of the mean flow downstream of a monopile tend to focus on the flow directly behind the monopile (at a distance between 2 and 15 monopile diameters, *D*)^30,31^, but not in the broader area behind the monopile. In the far-wake ( > 15*D* away), however, sediment suspension is still observed at scales up to tens of kilometers from even mature offshore windfarms^32–34^, which implies broader changes to seabed sediment transport pathways than the often-studied monopile scours. This potential alteration of seabed morphology and composition further away from the monopiles themselves has received less attention. A recent review of 314 pieces of evidence on the effects of offshore windfarms found no more than 10 studies on the effect of offshore windfarms on sediments, with most of those focused on sediment plumes and carbon storage^10,35^. In this context, we focus on the impact of the wake flow on the seabed beyond the near-field of monopiles.

The turbulent wake from a monopile represents a challenge for modeling approaches, which use the mean flow to calculate bed shear stress. This approach is the advised or default setting in many numerical models^36–38^. However, this approach will underpredict the bed shear stress in the wakes of these objects as the mean flow speed is lowered, but crucially, highly turbulent^39^. This leaves a major gap in many, if not all, predictions of bed shear stress around offshore windfarm foundations—and hence in identifying areas where the largest changes to seabed mobility can occur^25,28,29,40–42^.

To address this shortcoming, we demonstrate an approach that combines both the mean flow and turbulence to predict the magnitude and extent of changes to bed shear stress downstream of a monopile. We use this predictive capability to quantify changes to sediment transport capacity in a monopile wake. The ecological consequences of such enhanced sediment mobility are underreported and not well understood^35^, in particular beyond the near-field of scour-protected monopiles. Increased turbidity is a major concern for suspension feeders, and for predator-prey relationships^43,44^, while alterations to sediment composition can particularly affect benthic deposit feeders and burrowing infauna^45–47^. Changes in sediment composition from sand to gravel/rock, for example, through winnowing or erosion of the uppermost substratum layer, can fundamentally alter habitat suitability for many species^48–51^. Benthic community composition can further be affected by changes in bed morphology that alter substratum roughness and the accumulation of organic matter^52,53^.

Future offshore windfarms will cover even larger portions of the shelf, built in predominantly deeper waters (30 m +) that typically have lower bed shear stresses, and where sediments are mobilized less frequently^54^. This potentially renders habitats more sensitive to changes in bed shear stress and sediment mobility introduced by the next generation of deeper fixed-bed offshore windfarms. We therefore focus on that scenario via combined laboratory and modeling work that includes the important flow-sediment-morphology feedback processes in the wider area downstream of a monopile. These feedback processes can modify the turbulent wake, seabed morphology, and sediment composition. This complexity currently limits the advice given to industry and regulators on the implications of these recovery wakes and their effects on seabed substratum, and consequently habitats and the wider ecosystem. Better knowledge of the extent of seabed modification will inform the approach and the effectiveness of compensatory measures, both at a plan- and a strategic level if the effects are beyond the windfarm footprint. This knowledge will support advice on how to monitor these processes appropriately, linked to the possible impacts on key receptors. If certain seabed properties are particularly advantageous in a certain area for the ecosystem, this research can help identify the potential for nature-positive designs.

The questions we seek to address are: (1) What is the intensity and spatial scale of hydrodynamic effects on the bed from a scour-protected monopile when specifically accounting for turbulence, and not just mean flow? (2) What is the change in sediment transport capacity in that broader area away from the monopile when the seabed sediment is naturally immobile, but close to the threshold of mobilization? (3) How might accelerated seabed mobility affect seabed composition when the bed is already mobile?

In this work, we answer these questions with a suite of laboratory, numerical model, and field evidence. We create a large laboratory-scale model of a typical offshore windfarm monopile, with rock armor on a sandy sediment bed. The flow has been designed so that it could not mobilize the sandy sediments prior to encountering the monopile, but should mobilize that sand in the turbulent wake of the monopile. During the experiment, we measured the mean flow and turbulence downstream from the monopile and collected 3D topographic data of the bed before and after the experiment with a laser scanner at a resolution of 1 mm. Using this information, we create and validate a three-dimensional (3D) computational fluid dynamics (CFD) model using TELEMAC-3D, which includes rock armor and bedforms in the model bathymetry. We discern the 3D flow structure in the wake of the monopile and its impact on bed shear stress and sediment mobility, specifically comparing the relative contribution of turbulence and mean flow. In the field, we measure seabed topography, backscatter intensity, mean flow and turbulent kinetic energy in the wake of a monopile at an operational windfarm and demonstrate that turbulence is the driver of seabed modification from offshore windfarms.

## Results and discussion

### The 3D flow in the wake of monopiles enhances bed shear stress and can mobilize the seabed

**Fig. 1:**
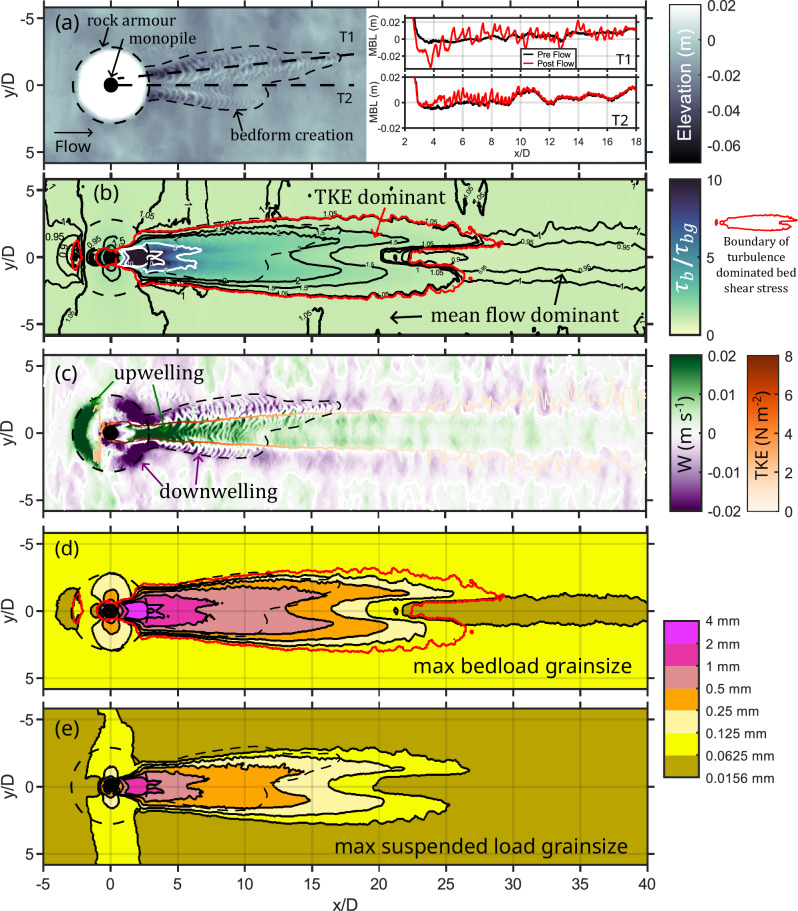
The effect of a monopile wakes’ turbulence on sediment transport capacity. **a** Laboratory LASER-scanned bed morphology, bedforms and the rock armor are outlined with long dashes. Inset are transects T1 & T2, which show the elevation relative to the mean bed level. T1 shows the bedforms formed over the pre-flow surface, and T2 shows the bedforms that form laterally to the monopile. **b** Shows the bed shear stress produced *via* the blended method from the validated TELEMAC-3D model, normalized by the background value of bed shear stress (0.1257 N m^*−*2^), color values maxed at 10 for visual clarity, maximum value is 24. Red line denotes the transition between mean flow and Turbulent Kinetic Energy (TKE) as the dominant source of bed shear stress. **c** Background color image shows the simulation’s vertical velocity averaged over the lower 20% of the flow depth, the two white to orange lines identify the horizontal location and intensity of the peak in TKE, either side of the monopile. **d**, **e** These are contour plots of the maximum grain size (in mm) which could be mobilized using the bed shear stress from the blended method. Dashed black line outlines the bedform field generated in the laboratory experiment. The red line denotes the transition between the mean flow and TKE as the dominant source of bed shear stress. Flow is left to right in all panels. Monopile diameter (D) is 0.25 m.

Two trains of bedforms emerged in the turbulent wake of the scour-protected monopile (Fig. 1a), extending along the downstream (*x*) axis of the flume. The bedform trains extended diagonally away from the centerline (Fig. 1a), one to 17 times the monopile diameter (*D*) downstream of the monopile (Transect T1–Fig. 1a), while the shorter train extended to 11*x*/*D*. Directly downstream of the monopile, bedforms exist to a similar length (11*x*/*D*) but wholly add to the bed elevation (Transect T2–Fig. 1a). The asymmetry in the length of the bedform trains could be caused by an asymmetry in the flow upstream of the monopile, or a small variation in the mobility of the sediment (e.g., due to compaction^55,56^). The maximum width of the bedform field, as measured in relation to the flume’s *y*-axis (perpendicular to the centerline), was 1.5 m (6*x/D*).

In our experimental flume setup, where the sandy sediment was not naturally mobile under the mean flow conditions created in the flume, only accounting for the wake effect on the bed in line with the monopile^30,31^ would have underestimated the length of the mobile bed by 35%. To what extent the presence of the bedforms and the rock armor modify the wake length in the centerline is unclear at present and needs further research. What is clear is that the wake effect on the bed from a realistic offshore windfarm is not wholly captured by just looking at the effect directly downstream of the monopiles – there is a strong lateral effect present which needs to be accounted for.

Here we use a blended approach to calculating bed shear stress, wherein the *mean flow method* commonly used to calculate bed shear stress was supplemented with the Turbulent Kinetic Energy (TKE) method (equation 9, Fig. 8), via selecting the higher of the two values (Eq. (17)). There is an excellent agreement between the measured and the modeled bed shear stresses (Fig. 8d) because this method combines the ability to calculate bed shear stress from turbulence as well as mean flow. The predicted bed shear stress from this blended method shows the wake of the monopile to be dominated by TKE, (Fig. 1b). The background bed stresses were measured to be 0.1257 N m^−2^ in an area upstream that was unaffected by the presence of the rock armor and monopile (Fig. 7d). Close to the monopile, and in the turbulent wake, the bed shear stress is amplified over the background level up to 24 times to 3.06 N m^−2^ (Fig. 1b). By 30*x/D* the bed shear stress due to the turbulent wake reduced to that calculated from only the mean flow (Fig. 1b), more than double the length scale measured in experiments elsewhere^30,31^, and 2.9 times longer than the predicted mean flow wake effect on the bed for this experiment from (Eq. (7)). The prediction of the length of the TKE wake is more uncertain, as the current curve (Eq. (8)) reduces asymptotically, and is based on data which shows no slope after 10*x/D*. Our experiment demonstrated that bed shear stress due to TKE from the wake reduced to values comparable to (or below) those of the bed shear stress driven by the mean flow between 20-30*x/D* downstream of the monopile.

The region dominated by bed shear stress from TKE alone (denoted by the red line) extends to 25*x/D* on average along the centerline and 6.4*x/D* in width perpendicular to the centerline (Fig. 1b). This region was not predicted to exist by the standard mean flow method of calculating bed shear stress (Fig. 6) and will have substantial effects on predicted sediment transport capacity. The width of the bedform field in the flume corresponds well with the steep drop-off in modeled amplification of bed stress. The extent of bedforms along the x-axis is smaller than the modeled extent of bed stress amplification, which in this direction drops off more slowly with distance away from the monopile, revealing the disparity in the length of the bedform trains and the conditions required to generate them. One such disparity may have been the time limitation on the laboratory run, which had to end after 12 h due to time constraints, since the bedform trains were still growing in length at the end of the experiment.

Figure 1c illustrates the processes that control near-bed turbulence, how they impact the spatial distribution of amplified bed shear stress, and their controls on bedform formation. Bedforms formed along the boundary between upwelling (green color scale) and downwelling (purple color scale) in the wake of the monopile, with the vortex cores slightly inside the region of upwelling. Bedform generation expanded laterally by two monopile diameters from the TKE vortex cores, which suggests that the downwelling process is important for bedform formation. In the upwelling region directly downstream of the monopile, bedforms were generated up to *11x/D* (Fig. 1a). The change between upwelling and downwelling in the flow coincides spatially with the transition in bedforms from a depositional (adding to bed level) to a more erosional character (Fig. 1a). Upstream of the monopile, the slope of the rock armor produces substantial upwelling before the flow reaches the monopile, demonstrating that the specific design of rock armor scour protection will influence near monopile flow structure. These 3D processes would not have been resolved with a computationally less expensive depth-averaged numerical model for the flow around a monopile, nor a 3D model that uses the hydrostatic approximation of the vertical velocity.

Our work demonstrates the effects of these complex monopile wake flow processes on sediment transport capacity. The area of the bed which has been modified by the turbulent wake flow is 8.158 m^2^, or 10.4 multiples of the monopile area (0.785 m^2^). This boundary is also plotted in Fig. 1d to demonstrate where the mean flow method and TKE method would predict changes to sediment transport. Inside the region of TKE dominant bed shear stress, a substantial increase in sediment transport capacity occurs, which can scour and/or modify seabed substratum up to 25*D* in the downstream direction and 6.4*D* in the across-stream direction (as per the grain size binning we have used). Whilst in the flume laboratory, the maximum grain size mobilized as bedload was 0.071 mm upstream of the monopile, the largest grains modeled in the wake to be mobile are 3.96 mm as bedload (Figs. 1d) and 1.9 mm as suspended load (Fig. 1e).

Sediment mobility due to changes in the mean flow (outside of the red line) primarily indicates changes in front of (lower mobility) and to the sides of the monopile (higher mobility)—with a long tail (evident only between 30–40*D*) of lower mobility from the reduction in mean flow speed downstream of the monopile. Whilst the absolute changes to sediment transport capacity predicted via the mean flow are small compared to those in the turbulent wake, the area they affect may end up larger than the area of the turbulent wakes, as the mean flow has been seen to accelerate between monopiles at the field scale, and the reduction in mean flow downstream of the monopiles can last > 40*D*^57,58^. Even larger-scale “blocking” of the flow by windfarms may well produce alterations to the mean flow speed in areas larger than the footprint of the windfarm^13^. This reiterates the importance of modeling the effects of both mean flow and turbulence for sediment transport. Our combined approach to calculating bed shear stress demonstrates how monopile wake flows can move sediments and alter seabed composition, with potential implications for ecological habitats in and around offshore windfarms^45^.

#### How the monopile 3D wake flow structure creates and defines the effect on the bed

**Fig. 2:**
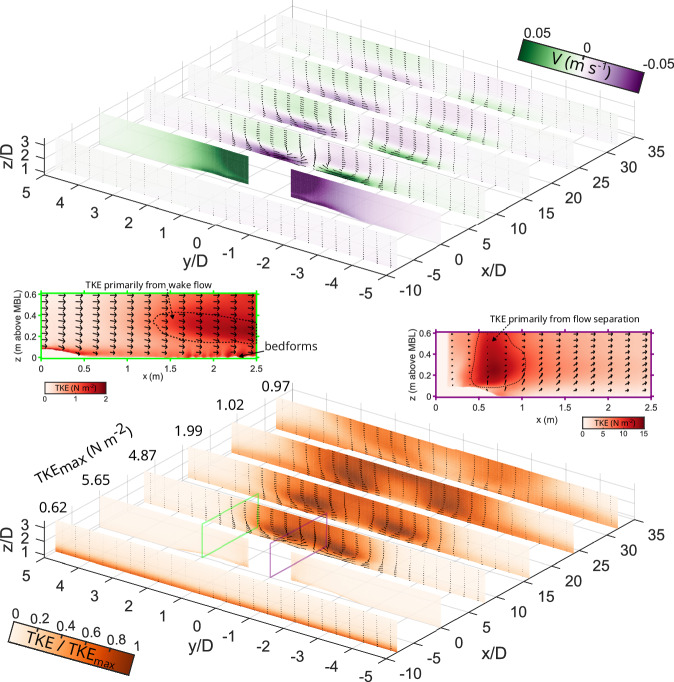
The three-dimensional (3D) structure of the turbulent wake. (top) Fence diagrams of the lateral velocity (V, m s^−1^) and (bottom) Turbulent Kinetic Energy (TKE, N m^*−*2^). Negative V velocities are flow directions towards negative y/D. Superimposed arrows show the time-averaged V and W flow directions, highlighting the secondary flow patterns in the wakes of the monopile. For visual clarity, these arrows have not been plotted in x/D = 0 as the lateral velocities were very large. Each plane of TKE has been normalized by the maximum value in each plane to better illustrate the spatial distribution of TKE, values for maximum TKE for each plane are given at y/D = 5. Cross sections of the TKE and U, W vectors are shown with purple (in line with monopile) and green (lateral to monopile) outlines.

The changes to vertical flow speeds and direction (Fig. 1c) illustrate part of a wider effect on the flow imparted by the presence of a monopile, i.e., the generation of two counter-rotating vortexes^19,23,27^. Our model results also show this complex three-dimensional wake flow structure (Fig. 2a), with two counter-rotating circulation cells in which flow moves away from the monopile (toward the outside of the channel) at the surface, and the flow moves towards the centerline near the bed (*x/D* = 0 to 30 Fig. 2a). This 3D wake flow structure is well known, but our results highlight the spatial linkage between the wake flow structure and the amplification of bed shear stress and alteration of bed morphology.

Flow separation in the monopile’s wake is generating the peak TKE value of 15 N m^−2^ over the sloping rock armor (Fig. 2 and insets), whilst TKE values further away peak at 4–5 N m^−2^ in the turbulent wake of the monopile ( > 5 *x/D*). It is this turbulence further away from the monopile that is driving the sediment transport responsible for the formation of the bedforms in the laboratory. This occurs where the lateral flow velocities switch direction in the vertical (Fig. 2, top panel), creating shear in the flow. The green inset plot at *y/D* = 2 in Fig. 2 shows how the downwelling flow and TKE combine in the same place over the long bedform train. The resultant three-dimensional wake flow generates near-bed turbulence that locally increases the shear stress over the threshold of motion, thus generating the bedforms.

The upwelling flow directly downstream of the monopile centerline causes the sediment plumes observed via satellites^32,33^, and the enhanced mixing of the flow, which has been shown to alter ocean stratification^6,9,12^. Both effects are actively researched for their potential impact on marine life. We show how the corresponding downwelling flow located laterally from the centerline of the monopile (which must exist to balance out the upwelling flow) can increase bed shear stress, cause erosion of the bed and generate bedforms (Figs. 1, 2). We demonstrate that the largest regions of wake flow-enhanced seabed mobility are not directly in line with the flow and the monopile, but laterally from that centerline. This is a feature not accounted for in present estimates of the wake length on the seabed^30,31^. The numerical models used at the field or shelf scale are typically not of sufficiently high resolution to capture this detail^12,58^, usually due to cost constraints. We argue that the 3D processes described herein also need to be accounted for in any assessment of changes to the seabed due to the installation (or decommissioning) of offshore windfarms.

The magnitude and extent of enhanced bed mobility shown in our laboratory experiment are defined by the fundamental physical processes that we described here. The flow –– sediment condition used in the experiment is designed to illustrate the effect of enhanced bed mobility induced by the turbulent wake. Offshore windfarms are constructed in locations where the natural state of the seabed could range from immobile to highly mobile, so the exact effect will be unique to every windfarm, and perhaps every monopile. The critical point we raise is that the monopile wake structure will enhance the bed shear stress with a greater magnitude and spatially larger extent than previously documented. Variability in the magnitude and extent of this enhancement may result from differences in the tidal range and tidal asymmetry. The exact impact on seabed sediments will depend on sediment sizes and substrate thickness. Spatially less extensive mobility would be expected for coarser sediments (e.g., gravels), with more extensive effects likely for finer (non-cohesive) sediments. Bedforms may reduce the 3D wake effect, as they produce their own turbulent wake flows which disturb larger-scale flow structures^59^. All these sources of variability can be considered with appropriate modeling as performed here.

#### Field demonstration of enhanced seabed stress and sediment mobility due to turbulent wakes from offshore windfarm monopiles

**Fig. 3:**
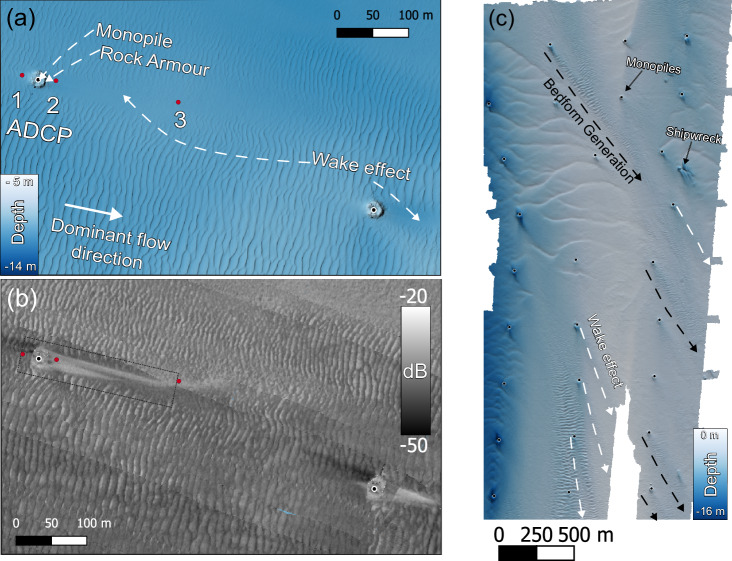
Field examples of the enhanced seabed mobility in the wakes of offshore windfarm monopiles. In **a**, **b** the Rhyl Flats offshore windfarm with 0.5 m resolution bathymetry (**a**) and multibeam echosounder (MBES) backscatter intensity (**b**) data collected in 2024 with location of the acoustic doppler current profiler (ADCP) deployment in 2023 (data in Fig. 4), and **c** the Scroby Sands offshore windfarm with 1 m resolution bathymetry data collected by CEFAS in 2006 (downloaded from www.marinedataexchange.co.uk/). Both digital elevation models are referenced to Lowest Astronomical Tide (LAT). In the Rhyl Flats windfarm, the background sediment transport regime is mobile and bedform producing, with flood tides dominant (West to East). In the wake of the two monopiles (monopile diameter, D = 4.7 m), bedforms were removed some 200 m (40D) away. An outline of the scale of the figures from the laboratory experiments is shown *via* the dashed black box, scaled up to the field. Scroby Sands windfarm, a more diverse range of monopile-wake effects is observed, with both bedform creation and removal shown.

To demonstrate that monopile wake effects on the seabed are indeed dominated by enhanced bed stresses many monopile diameters away, we first present seabed topographic and acoustic backscatter intensity data from a multibeam echosounder (MBES) survey at the Rhyl Flats offshore windfarm in 2024 (Fig. 3a, b). There is a strong tidal asymmetry at this site^60^ and a naturally mobile sediment regime with migrating bedforms. The monopile wake effect is in the dominant flood tide direction as evident in localized bedform removal more than 40*D* away (Fig. 3a), exposing acoustically more reflective gravels which underly the mobile sands at this site (Fig. 3b). The two lengths of exposed gravels have a spatial structure much like the 3D flow structure emanating from the monopile seen in the laboratory experiment and numerical model (Figs. 1, 2). Both bedform creation and removal are observed in the wake of monopiles at the Scroby Sands windfarm (Fig. 3c), depending on the local seabed mobility, but always far beyond the near-wake spatial extent. Our insights from flume laboratory experiments and our modeling approach lead the way to both measuring and modeling the exact impacts at these various sites, and allow for predicting monopile-induced wake effects.

**Fig. 4:**
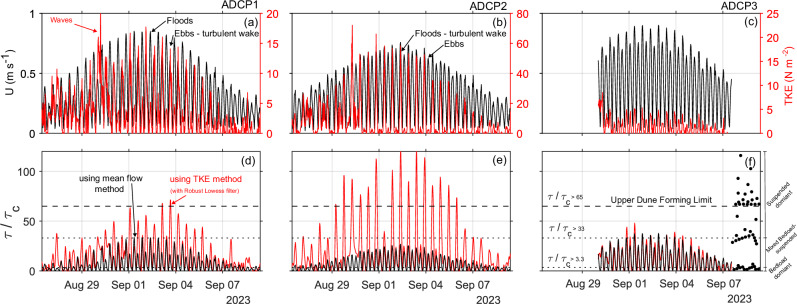
Comparison of mean flow and turbulence-derived bed shear stress from field surveys confirms the dominant role of monopile turbulence on sediment transport. Near-seafloor hydrodynamic monitoring data acquired using Acoustic Doppler Current Profiler (ADCP) data recorded between 26^th^ August 2023 and 9^th^ September 2023. Panels **a**–**c** display depth-averaged mean flow speed (Black) and depth-averaged median turbulent kinetic energy (TKE, Red) for ADCPs 1, 2 & 3. Panels **d**–**f** show bed shear stresses normalized by the threshold of motion for the 245 μm sand present at the site. These sediment transport thresholds are also plotted for comparison: bedload (${{{\rm{\tau }}}}/{{{{\rm{\tau }}}}}_{{{{\rm{c}}}}} < 3.3$), mixed bedload-suspended load ($3.3$ $> \,{{{\rm{\tau }}}}/{{{{\rm{\tau }}}}}_{{{{\rm{c}}}}} < 33$) and fully suspended load $({{{\rm{\tau }}}}/{{{{\rm{\tau }}}}}_{{{{\rm{c}}}}} > 33)$ as defined by^62^, and the upper limit of dune-forming conditions $\left({{{\rm{\tau }}}}/{{{{\rm{\tau }}}}}_{{{{\rm{c}}}}} > 65\right)$ as defined by^63^. Illustrative cartoons of the sediment transport regimes are included to the right of panel (**f**).

To demonstrate that the enhanced seabed stress is indeed due to enhanced turbulent kinetic energy in real monopile wakes offshore, we present water column data (Fig. 4) from three bottom-mounted, upward-facing, 5-beam acoustic Doppler current profilers (ADCPs) we deployed in 2023 around a monopile of the Rhyl Flats offshore windfarm (located in Fig. 3a).

To validate our method with offshore data, bed shear stresses were derived from the ADCP data over a large spring cycle via two methods: 1) a depth-averaged mean velocity method via e.q. 13–14, and 2) the TKE method (eq. 9), using data presented in Fig. 4a–c. To demonstrate how these bed shear stresses relate to sediment mobility (Fig. 4d–f), we normalize their values by the threshold of motion for the sandy sediment at this site (${D}_{50}$ = 245 μm^61^), using Eqs. (4–5), and compare with thresholds for various sediment transport regimes^62,63^. The flow conditions outside of the turbulent wake never reach the fully suspended or pass the upper bedform limit needed to remove pre-existing bedforms (Fig. 3a), but the calculated bed shear stress from the turbulent motion in the flow is repeatedly high enough to do this—particularly in the flood tide turbulent monopiles’ wake measured by ADCP2, where bed stresses calculated from TKE are 3–5 time higher than bed stresses calculated from mean velocity. In the ebb tide, where the monopile’s turbulent wake is measured by ADCP1, this bedform-removing threshold is only briefly reached, correlating with the observed spatial asymmetry of bedform presence either side the monopile (Fig. 3a). ADCP3 (Fig. 4c), was 40.8*D* away from the monopile and rarely measured the turbulent wake, despite evidence of a wake effect on the seabed at this location (Fig. 3a). Here, both methods of calculating bed shear stress show similar values, as would be expected for well-developed open channel flow^64^.

**Fig. 5:**
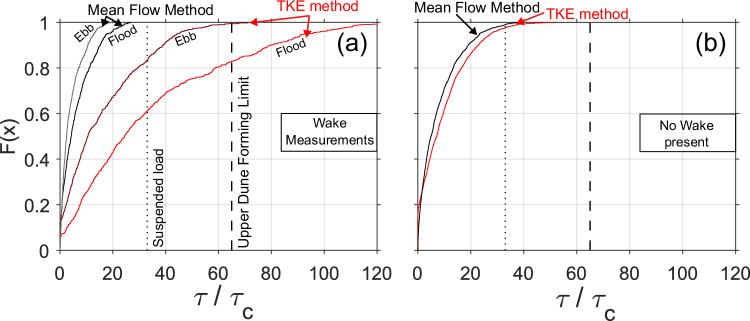
Cumulative frequency distributions of the ratio of bed shear stress derived from the ADCP data, over the threshold of motion for the sand at the Rhyl flats site, for the length of the deployment. **a** Shows bed stress ratios calculated using TKE from the data during the flood tide wake (ADCP2-floods) and ebb tide Wake (ADCP1-ebbs) and bed shear stress ratios from the mean velocity method. **b** Shows the similarity in methods when no turbulent wake is present. Sediment transport thresholds are plotted for comparison: fully suspended load $({{{\rm{\tau }}}}/{{{{\rm{\tau }}}}}_{{{{\rm{c}}}}} > 33)$ as defined by ref. ^62^ and the upper limit of dune-forming conditions $({{{\rm{\tau }}}}/{{{{\rm{\tau }}}}}_{{{{\rm{c}}}}} > 65)$ as defined by ref. ^63^.

The distribution of bed shear stresses across all measured flood tide wakes (Fig. 5a) via the TKE method was above the limit of dune-forming conditions 17% of the time, and 38.9% of the time in the suspended sediment regime. Bed shear stresses calculated via the depth average method for the same flows never reached the suspended regime. Due to the strong tidal asymmetry in this location, ebb-wake bed shear stresses via the TKE method only reached the upper limit of dune-forming conditions 0.6% of the time (Fig. 5a). Where the turbulent wake was not being measured (Fig. 5b), bed stresses calculated using the two different methods produced similar values, with values using the TKE method higher due to the implicit incorporation of wave bed shear stress.

#### Potential shelf-scale implications for habitat composition, distribution and ecological interactions

To illustrate the implication of the potential extent for seabed impact, we apply our laboratory findings to two hypothetical offshore windfarm designs where tidal flows, waves and sandy seabed substrate conditions are similar for each case, but where sizes of monopiles are either 5 m in diameter, representing early developments or 15 m, representing future developments (Supplementary material: Windfarm Area Calculations). Depths for the two windfarms scale with the monopile to keep the monopile diameter to depth ratio constant. Using our laboratory results as a guide rather than the field evidence (as that has multiple sediment classes producing its effect), we defined an area to be affected by the monopile wakes as 25 monopile diameters downstream and 6 monopile diameters laterally, and assumed this effect applies to either side of the monopile (symmetrical tides). In this hypothetical case, considering monopiles and rock armor as the main alteration of the seabed^55^, the infrastructure represents less than 1% of a windfarm’s area. The area exposed to enhanced seabed mobility at the scale shown in our laboratory experiment would cover 3% of a windfarm area with monopile diameters of 5 m in shallow waters, and 8% of a windfarm area with monopile diameters of 15 m in deeper waters—even though the latter windfarm would have fewer monopiles. The area of amplified bed shear stress produced in our laboratory experiment represents one hydro-sedimentary condition across the spectrum of tidal and sedimentary regimes. The exact effects on any windfarm in the world will depend on local hydrodynamic and sedimentary conditions, which require modeling validated with local observations. Factors such as Reynolds number and roughness of the natural seabed and/or scour protection are likely to scale the area affected, as there are phenomenological similarities with monopile scour^20^.

To demonstrate the potential scale of the effect of accelerated seabed mobility due to monopile wakes, we apply these calculations to the Northwest European shelf for existing and planned windfarms with the end members from the above hypothetical, but realistic, cases. Windfarm areas from https://emodnet.ec.europa.eu were filtered for overlaps between areas of planned, approved and under-construction windfarms, as well as removing tidal energy sites and cable routes from the dataset and any windfarm with an average depth deeper than 70 m, as these would be unlikely to use a fixed foundation design. This results in a total offshore windfarm area of 44,155 km^2^ for this region. Based on our results, the potential shelf area which could be affected by enhanced bed shear stresses from monopile wake flows is then predicted to be between 1325 and 3532 km^2^, which represents between 0.11 and 0.30 % of the shelf ( < 200 m deep). Another region undergoing rapid industrialization is the coastline of China, which is currently the largest offshore wind development region^65^, accounting for 80% of all installations in 2022^11^. China currently hosts the largest proportion of offshore wind capacity globally, with the area zoned for development between 2011–2020 being 10,401 km^2^, with further growth planned^66^. Based on our results a maximum potential area of 312 km^2^ to 832 km^2^ could be affected by the 3D wake flow enhanced bed shear stress from that generation of windfarms, although it should be noted that along the Chinese coastline the effect we describe may be limited to 20 m water depths, as jackets, tripods or multi-piles with a cap are generally the preferred foundation designs at water depths of 20–50 m^65,67^. Information on exact foundation design for each individual windfarm is often only available from proprietary sources, so we do not attempt to modify these estimates per foundation design and per windfarm, but rather seek to demonstrate the potential scale of the effects we have quantified.

As demonstrated in our work, laboratory experiments, numerical modeling and field observations are required to quantify the exact scale in time and space of the impact on the seabed and water column (Figs. 3, 4). The spatial scale of the impact can be larger than observed in a laboratory (Fig. 3), but also smaller. A key result presented here is that the effect on a sediment bed was far beyond scour^23^ and edge scour^27^, and it created a broader change to sediment mobility, many multiples of the monopile diameter downstream and laterally from any single monopile. Tidal currents, bed stress and the related sediment characteristics are major drivers of benthic assemblage composition in shelf environments, alongside temperature and depth^45,68^. Therefore, if offshore windfarms can change the seabed and sediments, there may be an associated effect on marine life from these windfarms. Changes to bed shear stress can change benthic faunal assemblages, including their functional composition^69^. The creation of bedforms can add heterogeneity to sediment distributions, with bedform lee sides accumulating fines and providing shelter for infaunal species^52,53,70^. Enhanced bed shear stress will likely increase the median grain size in the region affected within the sediment wake due to the selective entrainment of sediments out of the bed^62,71^.

The enhanced bed shear stress emanating from each monopile could create areas with a different substratum (Fig. 3b), and therefore benthic assemblages, and the overall habitat heterogeneity in the wider area may change, resulting in a patch mosaic of habitat types. This could influence beta- (between patches) and gamma- (overall) diversity, and may influence overall species connectivity in the ecosystem^72,73^. Patch mosaics will benefit those species that require multiple habitat types in their direct surroundings, while they may hinder those that need large, contiguous areas of one habitat type for their survival^74^. How such effects would develop in specific windfarms depends on the original seabed substrate, hydrodynamic regime and habitat distribution, as well as other potential factors of influence, such as reef effects created by the infrastructure itself and associated changes in organic matter input or the removal of other anthropogenic pressures, e.g., fishing^75^. Disentangling such effects will require specific and long-term monitoring of the benthic ecosystem in and around offshore windfarms, including detailed mapping of habitat distribution and quantitative characterization of the different biotope components (in- and epifauna), evaluated against the various interacting environmental drivers and anthropogenic impacts. Such work often happens at the planning stages of offshore windfarms, but less frequently in a more targeted fashion after commissioning.

Changes to the seabed substratum, turbidity and turbulence in the water column could alter the behavior of important prey species that are sensitive to fluctuations in sediment composition. Sand eels (*Ammodytidae*), for example, are very sensitive to the amount of fines (silt and clay) in the sand, tolerating no more than a weight fraction of 6% in the sediment^76,77^. Winnowing of fines from the upper sediment layer via increased turbulence in the water column may result in a more suitable habitat for sandeel populations^78^. Alternatively, if too much sand is stripped away, the proportion of gravel may increase, and therefore the habitat may become unsuitable^51^. Overall, any redistribution of important sandeel habitat due to changing sediment composition is likely to have consequential effects on their predators.

Seabird species that follow plunge-diving strategies based on aerial detection and ambushing of prey (i.e. *Sterna*) could select more turbulent^79–82^ and turbid waters^43,83,84^. By contrast, seabirds with pursuit-diving strategies based on underwater detection and active pursuit of prey (i.e., *Phalacrocoracidae*) could prefer less turbulent^85^ and clearer waters^86–88^. These examples illustrate the potentially contrasting effects that the monopile wake flow could have on marine life, and the need for a holistic view when investigating the impact of this infrastructure. The ecologically relevant changes to sediment mobility as we predict here should thus be considered when environmental impacts and opportunities for biodiversity compensation are investigated.

Our research also delivers insight into the potential effects of decommissioning and removal of offshore energy infrastructure from the seabed once it has reached the end of its serviceable life^89^. Removing such infrastructure, and thus returning the seas to their prior “natural” state (which is often required by law, e.g., following OSPAR Decision 98/3), might not always be the best option for marine ecosystems^90,91^. So far, the primary focus of the discussion has been on the role of infrastructure as artificial reefs^92^ and the resulting mix of what some perceive as desirable (e.g., enhanced diversity) and undesirable (e.g., a vector for invasive species) ecological consequences. Here, we show that alteration to seabed mobility, and potential for seabed modification, which can change the distribution of benthic habitats, are additional potential effects associated with the presence (or removal) of infrastructure, and need to be taken into account when designing nature-positive approaches to marine infrastructure^3,4^.

Better forecasts of seabed sediment mobilization^93^ and the extent to which it can alter the distribution of benthic habitats can strengthen marine spatial planning. The work presented here will help in the development of robust, multi-scale quantifications of seabed sediment dynamics in places where new morphodynamic feedbacks are introduced by seabed infrastructure, which we show can amplify bed shear stress and resuspension well beyond the immediate scour zone. We advocate for our method of integrating field observations and process-based models as a robust way to anticipate environmental thresholds with confidence and guide evidence-based marine spatial planning to safeguard carbon-rich seabed sediments and their habitats, and the infrastructure we increasingly rely on.

**Fig. 6:**
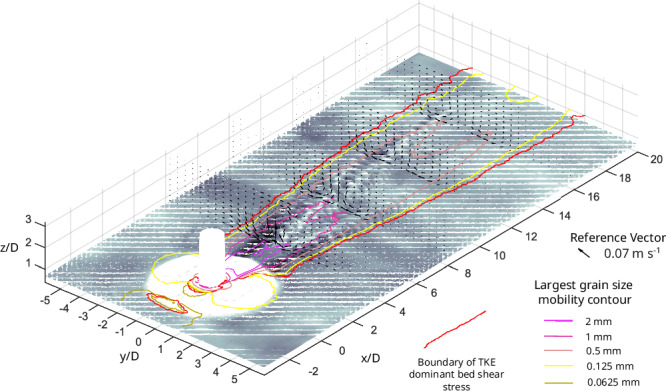
Summary diagram which combines the laser scan of the laboratory bed, the lateral and vertical flow vectors derived from the numerical model, and contour lines of maximum grain size able to be mobilized by the modeled bed shear stress. The turbulent wake eminating from a monopile both enhances bed shear stress and can mobilise sediment, the shape of effect on the bed is defined by the three dimentional structure of the wake.

As the world’s shelf seas continue to be industrialized, particularly for offshore wind energy, there is a need to be able to predict the changes to the seabed caused by this infrastructure. We have combined laboratory, three-dimensional numerical modeling, and field observations to demonstrate that turbulent wakes enhance sediment mobility in the wake of a scour-protected monopile. Our laboratory case study (summarized in Fig. 6) represented a seabed with sediments that are naturally immobile, much like those of many of the planned large windfarms, and the results demonstrated how the turbulence generated in the wake of the monopile can enhance bed shear stress, creating regions of accelerated sediment mobility beyond what has been previously predicted. We demonstrate the potential for a broad-scale increase (at least to 25 monopile diameters (*D*) away) in sediment mobility. The spatial scale and shape of this enhanced sediment mobility are defined by the three-dimensional mean flow structure produced in the turbulent wake of the monopile. Two regions of peak bed shear stress were focused into two lines located 2–3*D* either side of the monopile centerline, controlled by the presence of two counter-rotating vortexes in the monopile’s wake. This spatial pattern has been confirmed in the field. The length scale of this enhanced sediment mobility in the laboratory experiment was 17*D* away from the monopile, whilst the results from the computational model show bed shear stress amplifications to 25*D* away, 3 times longer than previously predicted, and 6.4*D* laterally.

Our method for predicting seabed modifications in the wake of windfarm infrastructure uses a bed shear stress derived from near-bed turbulence when it is dominant over the mean flow, and we quantify the so far underestimated magnitude and spatial extent of a wake effect where the turbulence is the dominant factor in enhanced sediment mobility. We suggest that this enhanced sediment mobility may affect benthic communities with potential consequential impacts for higher trophic levels, and our methods can help consider environmental impacts and opportunities for biodiversity compensation for marine spatial planning related to any seabed infrastructure project.

## Methods

### Laboratory work

**Fig. 7:**
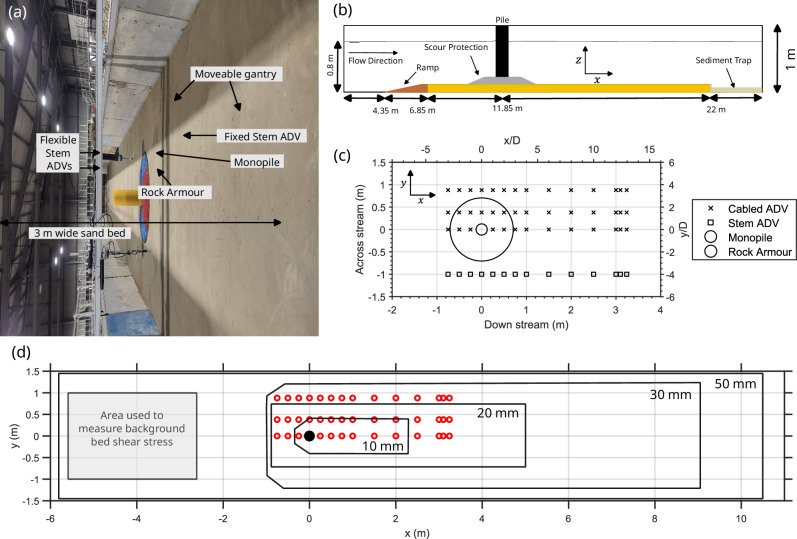
Overview of the laboratory experiment. **a** A photograph of the laboratory setup prior to filling the tank, view is from the top of the ramp looking downstream. **b** Cross-sectional diagram (not to scale) of the laboratory setup. **c** Shows the plan view of the Acoustic Doppler Velocimeter (ADV) measurement locations, with both geometric (bottom and left axis) and those normalized by monopile diameters coordinates (top and right). **d** Displays the model boundary, and refinement regions, as well as the ADV locations used in the model validation, and the area upstream of the monopile used to calculate the background value of bed shear stress.

The UK Coastal Research Facility at HR Wallingford is a 55 m long by 27 m wide recirculating wave-current basin^94^. In the basin, a 3 m wide channel was built using tank plates secured to the floor of the basin and sealed using a rubber membrane. A moveable 4 m long gantry was installed onto the tank plates to hold instrumentation (Fig. 7a). Our type-site of a 4.7 m diameter monopile in 12 m depth became a monopile of 0.25 m in 0.6 m of water which, with the 0.2 m of sediment thickness needed to ensure scour to the flume base was prevented, kept the experiment within the flow depth limitation of the basin (0.8 m). The monopile was secured to the floor of the basin at 5 m along a 15.15 m long sediment bed (Fig. 7b). The geometric scaling ratio between field and lab is therefore 1:18.8 with a monopile diameter to flow depth ratio of 2.4, which is typical of a shallow water monopile design^41,95^. At the upstream end of the channel, a 2 m long ramp with a slope of 1:10 was installed to transition the flow and constrain the sediment bed. A 0.5 m long transition section was installed between the top of the slope and the start of the sediment bed to prevent scouring of the sand at the top of the ramp. Downstream of the sediment bed, a sediment trap was constructed from blocks to prevent the loss of sediment into the basin sumps.

The rock armor design was based on two parameters: firstly, that the rock should not be mobilized around the pile wall by the flow during testing, and secondly, the thickness and lateral extent of scour protection should be representative of realistic field rock armor design. A high-density rock (3050 kg/m^3^) with a median diameter *D*_*50*_ of 12 mm was used to represent rock armor. The surface of each quadrant of the rock armor was spray-painted to identify any points of failure (Fig. 6a). During testing, the rock at the pile wall was observed to be stable.

The recommended top extent of the armor layer is 3 to 4 times the pile diameter, excluding the additional rock volume to form a falling apron with edge scour formation^95^. We optimized this based on the experience of similar testing to 2.7D and to provide sufficient space in the flume width to not create artificial lowering of the sand bed between the rock and the walls of the flume. The lateral extent of the rock from the pile wall was set to 1.85 times pile diameter at the sediment level, or total bottom width 4.7D. A single-layer scour protection design was chosen, where one grade of sediment is placed to provide both the filter and armor function required in the scour protection^96^. The thickness of protection was 0.084 m, i.e., 7*D*_*50*_, and the installed perimeter slope of the scour protection was 1:3, which is representative of offshore designs.

The choice of sediment size used to represent the bed was based on achieving similarity in the threshold of motion and settling velocity of the sediment between prototype and model scale. The mobility of the sediment (${\theta }_{{SF}}^{{\prime} }$) was estimated using a 2D depth average approximation using the method of^97^

1 $${\theta }_{{SF}}^{{\prime} }=\frac{{\rho }_{w}{\bar{U}}^{2}}{\left({\rho }_{s}-{\rho }_{w}\right){({C}^{{\prime} })}^{2}{D}_{50}}$$

2 $${C}^{{\prime} }=18\log \left(\frac{4h}{{D}_{90}}\right)$$

where ${\theta }_{{SF}}^{{\prime} }$ is the skin friction shields parameter), $\bar{U}$ is the depth averaged velocity, ${C}^{{\prime} }$ is the Chezy coefficient for roughness, ${D}_{50}$ and ${D}_{90}$ are the 50th and 90th percentiles of the grain size distribution, $h$ is the flow depth, ${\rho }_{s}$ is the density of sediment and ${\rho }_{w}$ is the density of the water.

The fall velocity of the sediment was estimated using the method of^98^:

3a $${w}_{s}=\frac{{Rg}{D}^{2}}{{C}_{1}\nu+\left(0.75{C}_{2}{Rg}{D}^{3}\right)}$$

3b $$R=\frac{{\rho }_{s}-{\rho }_{w}}{{\rho }_{w}}$$

where *g* is the acceleration due to gravity, $\nu$ is the kinematic viscosity of the water, and $D$ is the grain size. values of ${C}_{1}$ = 18 and ${C}_{2}$ = 1 for the constants, nominal for the normally graded sediments used herein.

The theoretical threshold for initiation of sediment motion was calculated using the modified Shields curve of^99^, with the non-dimensional particle diameter:

4 $${D}^{*}={{D}_{50}\left(\frac{\left(S-1\right)g}{{\nu }^{2}}\right)}^{1/3}$$

Where S is the specific gravity of the sediment. The initiation of sediment motion was calculated via:

5 $${\theta }_{{crit}}^{*}=\frac{0.30}{1+1.2{D}^{*}}+0.055\left(1-{\exp }^{-0.02{D}^{*}}\right)$$

and suspension via^100^

6 $${\theta }_{{sus}}^{*}=\frac{0.30}{1+{D}^{*}}+0.1\left(1-{\exp }^{-0.05{D}^{*}}\right)$$

Typical hydrodynamics and sediment at prototype scale are presented in Table 1 along with equivalent parameters at model scale.

**Table 1:** Laboratory flow conditions, with a field-scale example

	*Depth (m)*	$\bar{U}$ *(m s*^*−1*^*)*	*Re*	*Fr*	Strouhal number	*D*_50_, *D*_50_ (μm)	*ρ*_*s*_ (*kg* *m*^−3^)	${\theta }_{{SF}}^{{\prime} }$	${D}^{*}$	${\theta }_{{crit}}$	${\theta }_{{sus}}$	${\theta }_{{SF}}^{{\prime} }/{\theta }_{{crit}}$	${w}_{s}$ *(m s*^*−1*^*)*
Field Example	12.7	0.34	3.62 × 10^6^	0.03	0.19-0.2	245, 290	2650	0.028	6.188	0.042	0.068	0.67	0.030
Laboratory	0.6	0.24	1.3 × 10^5^	0.1	0.17	275, 290	2650	0.024	6.259	0.042	0.068	0.58	0.039

### Prediction of near-bed wake length before the experiment

Following^31^ we estimated the near-bed wake length to be 2.15 m (8.6*D*) using:

7 $$x/D=(14.1-22.9{U}_{c\infty })$$

Where ${U}_{c\infty }$ is the free stream velocity which is unaffected by the monopile, and *x/D* is the number of monopile diameters downstream where the mean flow has returned to within 5% of ${U}_{c\infty }$ The reduction in near bed TKE downstream of the monopile can be described via the curve:

8 $$\frac{k}{{U}_{c\infty }^{2}}=\alpha+\frac{\beta }{{n}_{D}}$$

where $\alpha$ = 0.0119, and $\beta$ = 0.3366^31^. This curve is validated to 15*D,* but the data upon which it is based is asymptotic after 10*D* (2.5 m). Consequently, the measurement grid was adjusted to reach as far as possible downstream (limited by instrument communication lengths), to 3.4 m downstream of the monopile (i.e., 13.6*D*). Figure 7c, d shows the plan-view grid of measurement points where data was collected. This measurement grid was considered both sufficiently long and high density enough to capture the detail within the predicted wake lengths from the monopile and provide comprehensive validation data for the numerical model.

### Flume control

Flow speed was gradually increased from stationary to the target value over the course of 2–3 h, whilst sediment motion in the flume was carefully observed until bedform growth downstream of the monopile began, at which point the flow speed was not increased.

Once the desired flow conditions were achieved, flow depth and discharge remained constant for the experiment, which lasted 14−16 h for the data to be collected. Flow conditions in the flume were controlled by the flume’s pump control systems and water level meters in the basin. An automated fill program was able to fill the flume at a non-linear rate. This allowed the worked bed to be more slowly drained once the water level was close to the level of the sediment bed, reducing the potential of draining artifacts in the scans. Drain times post experiment were 12–16 h. Flow depth gauges were marked throughout the flume, and depths within the channel were set to 0.6 m ± 0.01 m and were recorded throughout the experiments.

### Velocity data collection

A mobile gantry was used to move an array of acoustic Doppler velocimeters (ADVs). A fixed stem Nortek Vector was positioned to the right (flow right) of the monopile and moved only in the X direction and measured at 80% of the flow depth (i.e., 0.48 m below the water surface), Fig. 7a, c. A gantry system containing a cabled Vector ADV and a Vectrino Profiler (ADVP) was fitted so that they could move in X and Y directions, and also be moved (via threaded screw) in the vertical (Z) direction.

ADVs were set to record at 16 Hz, with a 0.3 m s^−1^ velocity range. The Vectrino profiler was set to record in high-power mode at 50 Hz, with bin sizes of 2 mm, and a velocity range of 0.4 m s^−1^. Seeding material was not added to the flows, due to the possibility that the 2–4 µm scale material may affect the mobility of the sand^101^. As sediment mobility was the predominant variable in these experiments, it was deemed more important not to affect it. Natural seeding from sediment suspension and the facility’s borehole freshwater offtake supply provided sufficient natural scatterers.

ADV measurements were started once the bed was developed (4–5 h). Each point was measured for three minutes, resulting in a run time of approximately 12–14 h to complete the grid for each condition. Once data collection was complete, the flume was slowly stopped (20 min) and carefully drained for laser scanning.

### ADV data post processing

The ADV and ADVP data were filtered for low signal-to-noise ratio but not correlation, as highly turbulent flows can produce low correlation but still good data^102^. ADV data was filtered using the method of^103,104^, whilst the ADVP data was filtered by the method of^102^. Spurious data, such as bed echoes in the ADVP data, and low seeding conditions were removed. For the ADVP data, this often resulted in 2–4 data points where the instrument had the highest signal-to-noise ratio^105,106^.

### Laser scanning

A Trimble X7 Terrestrial Laser Scanner (TLS) was employed to capture three-dimensional elevation data of the mobile bed. Before each test, the mobile bed was scanned at ten locations along the flume length after placing the mobile sand bed and rock armor, and again at the end of the test. The scans cover an area of 75.7 m^2^, including the area from 5 m upstream of the monopile to 10 m downstream, with on average 1 million points per square meter. The point clouds captured by the TLS were processed using Trimble Realworks (version 12.3). The digital elevation models produced are of 1 mm resolution. The bedform detection tool BAMBI^107^ was used to measure bedform geometry from the bed scan, and mean ripple heights ranged between 0.005 m and 0.015 m, with mean lengths a more constant 0.15 m. Lee side angles measured between 25 and 35 degrees.

### Data collation and calculation of bed shear stress

Once filtering was complete, mean flow and turbulence data were produced for the 3D grid of measurement points. Where repeated measurements were collected, the average of each collection was taken. Given the complex flow in the channel, estimating the bed shear stress via a Law-of-the-Wall or depth-averaged approach was deemed inappropriate. Theoretically, the Reynolds stress method should be the most accurate^108,109^; however, as exact control of the orientation of the instrument could not be assured, Reynolds stresses were not calculated as the error increases by at least 10% per 1 degree of angle away from the horizontal^110^. Therefore, as it has been shown to produce both accurate and precise values by numerous publications^110–114^, the turbulent kinetic energy method was used^115^, via:

9a $${TKE}=0.5\rho (\bar{{u}^{{\prime} 2}}+\bar{{v}^{{\prime} 2}}+\bar{{w}^{{\prime} 2}})$$

9b $${\tau }_{b}=0.19{TKE}$$

where the mean of the velocity fluctuations was taken at the measurement height above the bed that was closest to 10% of the flow depth, which meant it was located within the constant stress layer.

### Numerical model

As the laboratory data only covered 25% of the experimental flume area, a numerical model was used to gain insight into the entire domain. TELEMAC-3D is an open-source, three-dimensional, unstructured, finite element code solver for free surface flows, solving the Reynolds-averaged form of the three-dimensional Navier-Stokes equations^116^. Here, we used the code in its non-hydrostatic mode. In this mode, the hydrostatic components are initially calculated, before the Poisson pressure equation is solved for the non-hydrostatic component and used to correct the velocities and ensure a divergence free velocity field^117^:

10 $$\frac{\partial u}{\partial x}+\frac{\partial v}{\partial y}+\frac{\partial w}{\partial z}=0$$

11 $$\frac{\partial u}{\partial t}+u\frac{\partial u}{\partial x}+v\frac{\partial u}{\partial y}+w\frac{\partial u}{\partial z}=-\frac{1}{\rho }\frac{\partial p}{\partial x}+\nu \Delta \left(u\right)+{F}_{x}$$

12 $$\frac{\partial v}{\partial t}+u\frac{\partial v}{\partial x}+v\frac{\partial v}{\partial y}+w\frac{\partial v}{\partial z}=-\frac{1}{\rho }\frac{\partial p}{\partial y}+\nu \Delta \left(v\right)+{F}_{y}$$

13 $$\frac{\partial w}{\partial t}+u\frac{\partial w}{\partial x}+v\frac{\partial w}{\partial y}+w\frac{\partial w}{\partial z}=-\frac{1}{\rho }\frac{\partial p}{\partial z}+\nu \Delta \left(w\right)+{F}_{z}$$

14 $$p={p}_{{atm}}+\rho g\left({Z}_{s}-z\right)+{\rho }_{o}g\int _{Z}^{{Z}_{s}}\frac{\Delta p}{{\rho }_{o}}{dz}+{p}_{d}$$

where $u$, $v$ and $w$ are the three-dimensional components of velocity; ${F}_{x}$, ${F}_{y},{F}_{z}$ are source terms and $\nu$ is the effective viscosity that needs to be computed by a turbulence model. The pressure is calculated in the last equation, where ${\rho }_{o}$ and $\Delta p$ are the reference density and the variation of density, respectively, and ${Z}_{s}$ is the free surface elevation.

The one-equation Spalart-Allmaras turbulence model^118^ was used as it has been demonstrated to be an efficient and accurate model for flows with adverse pressure gradients, particularly flows around monopiles and bedforms^19,119,120^. In this model the eddy viscosity, ${\nu }_{t}$, isn’t solved directly Instead, a transport equation for a working variable $\widetilde{\nu }$ is solved. From this, the eddy viscosity is computed:

15 $${\nu }_{t}=\widetilde{\nu }{f}_{v1},\quad {f}_{v1}=\frac{{\chi }^{3}}{{\chi }^{3}+{C}_{v1}^{3}},\quad X=\frac{\widetilde{\nu }}{\nu }$$

where ${f}_{v1}$ is a damping function that limits the turbulent viscosity near solid walls, and ${C}_{v1}$ = 7.1.

“Smooth” boundaries in the model, such as the monopile and the sidewalls, have Dirichlet boundary conditions so that ${\widetilde{\nu }}_{{wall}}=0$. Turbulence properties in the nodes adjacent are calculated via the turbulence model based on the velocity gradient, which is calculated using a Reichard law, assuming the first node away from the boundary is in the log-layer.

For hydrodynamically “rough” boundaries in the model (the sand and gravel on the bed), roughness is introduced using the equivalent (Nikuradse), sand grain roughness^121^. The roughness values were calculated using the Van Rijn estimate^122^ of grain roughness for the sediments (3D_90_) used in the laboratory (sand roughness length ${k}_{s}$ = 0.00087 m, gravel ${k}_{s}$ = 0.036 m). Velocities at rough boundaries are calculated using the law of the wall, using the roughness lengths specified on the bottom boundary. The model, therefore, assumes the first node above the boundary is within the log-layer. For the Spalart-Allmaras turbulence model^118^, TELEMAC 3D uses an effective wall distance:

16 $${d}_{{eff}}=d+{C}_{t4}{k}_{s}$$

Where $d$ is the distance to the wall, ${k}_{s}$ is the sand grain equivalent Nikuradse roughness, ${C}_{t4}$ is 0.5. The distance ${d}_{{eff}}$ replaces $d$ in the destruction term in the turbulence model to mimic the loss of the viscous sub-layer in rough wall flows. Several other constants are used in this turbulence model, all of which are the same as the original publication except: $\sigma=0.85$, ${C}_{b1}=0.105$, ${C}_{w1}=2.07$, which have been found to perform better in TELEMAC 3D^123^.

Flow was discretized using an unstructured triangular mesh, created in *BlueKenue*^124^. A domain covering a sandy section of the flume experiments (3 m × 16.2 m) was created with a resolution of 50 mm (Fig. 7d). Various monopile grid resolutions were tested, and the best results were obtained with the highest resolution, 2.45 mm (see supplementary Table S1). Refinement regions upstream and downstream of the monopile increased the mesh resolution from 10 mm to 30 mm in the wake, over the bedforms and rock armor. The 10 mm near-field region was found to be essential to reduce numerical diffusion in the monopiles’ turbulent wake.

Wall-distances $({y}^{+}=y{u}^{*}/\nu )$, values for the monopile on average are 47, to a maximum of 140. Low values (minimum of 3.1) are found in the flow separation bubbles. Whilst ${y}^{+}$ values this low reach into the viscous sub-layer, the low velocity magnitudes ( < 0.05 m s^−1^) in these regions should result in little error due to overprediction of shear stress on the wall. Where velocities at the monopile surface are high, ${y}^{+}$ values are between 40–140.

The model used a timestep of 0.002 s to keep the Courant number under 0.5, ensuring model stability. Models were run for 560 s, with data recorded at 5 Hz for the final 3 min of the model run. For advection, the locally semi-implicit predictor-corrector scheme (LIPS) was used^125–127^. As the solution was unsteady in time, the option of three sub-steps for corrections to distributive schemes was used, and implicitation for depth and velocity was set to 0.5.

The conjugate gradient method was used to solve the diffusion of velocities, propagation, Poisson pressure equation, and the diffusion of TKE production and dissipation, epsilon. Diagonal preconditions were used for all except for the diffusion of velocities and the Poisson pressure equation, where a cumulated diagonal preconditioning with a direct solution along the vertical direction was used. This approach was found to substantially improve computational time over the default settings of the model. Solver convergence criteria were set to 1 × 10^−8^, with 1 × 10^−10^ for the diffusion of velocities.

Flow depth and discharge were specified at the inlet (upstream boundary), with a power law for the velocity profile specified in the vertical. The flow depth inlet was fixed at 0.6 m high with a discharge of 0.435 m^3^ s^−1^. The outlet (downstream boundary) was given a fixed discharge of −0.435 m^3^ s^−1^, and a free depth to account for the water surface slope.

### Calculation of bed shear stress and maximum grain size in the numerical model

**Fig. 8:**
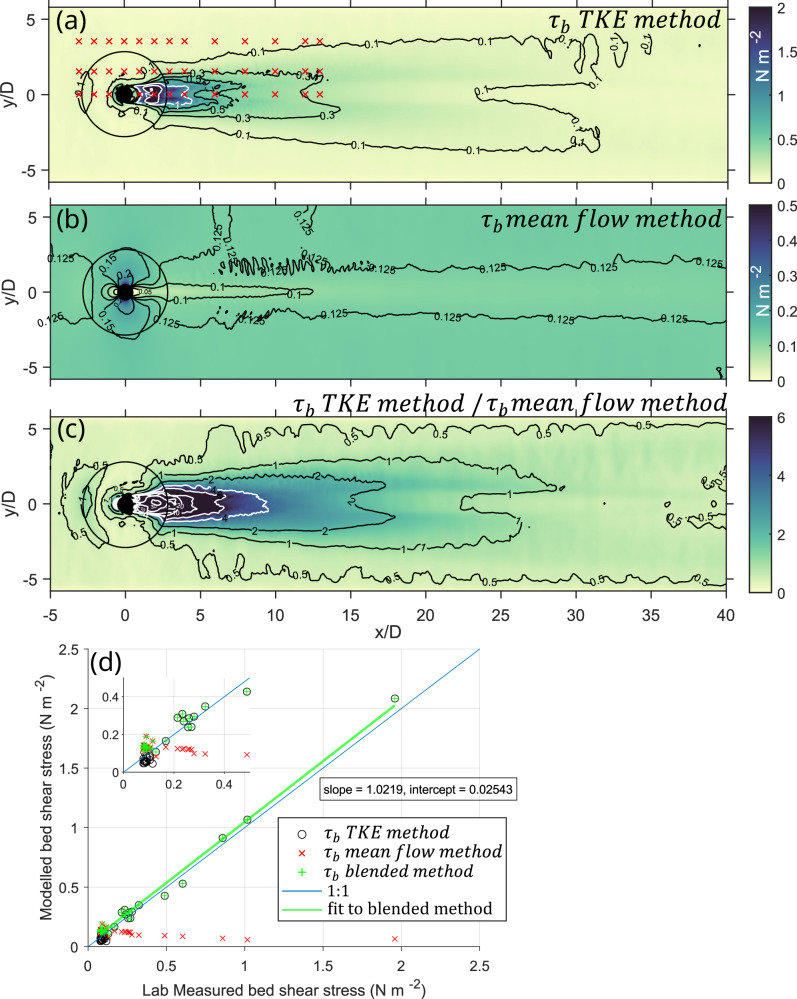
Comparisons of mean flow and turbulent-derived bed shear stress from the laboratory experiment and numerical model. **a** Plot of the bed shear stress from the model as calculated *via* the turbulent kinetic energy (TKE) method. Red x’s denote the locations of bed shear stress measurements in the laboratory experiment. Black circle outlines the rock armor. A solid black circle denotes the monopile. Flow is left to right. **b** plot of the bed shear stress as calculated from the mean flow method, note the change in magnitude compared to the plot above. **c** A comparison (*via* ratio) of the two methods of calculating bed shear stress. **d** A regression against laboratory calculated bed shear stress for the three methods of calculating bed shear stress from the numerical model, with an inset figure focusing on the lower values.

A 2D depth-average approximation using the mean flow velocity, depth and bed roughness (which we describe as the *“*mean flow method*”*) is likely to be unreliable in the turbulent wake of an object^110–112,114^, particularly monopiles^39^. The amount of turbulence produced by the monopile renders the Law-of-the-Wall or quadratic stress methods of calculating bed shear stress unsuitable, as they assume the mean flow is the driver of near-bed turbulence. As such, to calculate bed shear stress, we use the same method as used on the laboratory data (equation 9), which we call the *“*TKE method*”*. However, in the model, there are two sources of TKE: one from the turbulence model^118^, but also from the resolved eddies. Here we combined these two values as this gives the true value for TKE in the model – both modeled and resolved. In Fig. 8, we compare these two methods of calculating bed shear stress from the model data (Fig. 8a–c). This shows that the TKE method correctly predicts the high bed shear stresses in the wake of the monopile, but the mean flow method correctly represents the “free” or undisturbed flow, which is where mean flow is dominant. The *“*blended method” shown in Fig. 1b combines the values of bed shear stress from the TKE method and the mean flow method by taking the highest value of either method at each location:

17 $${\tau }_{{blended\; method}}={{{\rm{MAX}}}}({\tau }_{b\,{mean\; flow}},{\tau }_{{bTKe\; method}})$$

The boundary between TKE-dominant bed shear stress and mean flow dominant bed shear stress is indicated by the contour line with a value of 1 in Fig. 8c.

Comparison of the TKE method, the mean flow method and the blended method shows that the TKE method alone compares very well against the laboratory values of bed shear stress. The inclusion of values from the free stream via the mean flow method into the blended method improves the fit particularly at the low end of the range of bed shear stresses (Fig. 8d). The blended method gives a coefficient of determination of 0.99, slope of 1.02, intercept of −0.025 N m^−2^, and a root mean squared error of 0.042 N m^−2^, providing a satisfactory degree of confidence to the bed shear stresses produced from this method.

Following this validation, the Shields value ($\theta$) was calculated (using the blended method) for a range of grain sizes from 0.001 mm to 40 mm (in steps of 0.002 mm) via:

18 $$\theta=\frac{{\tau }_{b}}{\left(\left({\rho }_{s}-\rho \right)g{D}_{50}\right)}$$

Values for the threshold of motion and suspension (Eqs. (5, 6)) were also calculated for this range of grain sizes. The non-dimensional Shields values were then calculated for bed load ${\theta }_{{crit}}=\,\theta /{\theta }_{{crit}}^{*}$, and suspended load ${\theta }_{{sus}}=\,\theta /{\theta }_{{sus}}^{*}$, the maximum grain size which could be mobilized for each point was then the closest value to unity in ${\theta }_{{crit}}$ and ${\theta }_{{sus}}$.

The eddy shedding frequency in the numerical model was measured at 1.5 m downstream of the monopile (*x/D* = 6) and at the free surface. A dominant frequency of 0.166 Hz was found, which, for a mean flow of 0.24 m/s, and a pile diameter of 0.25 m, produces a Strouhal number of 0.173 in excellent agreement with prediction.

#### Field data collection

A Norbit Winghead i77 was used to collect the Multibeam Echosounder (MBES) bathymetry and backscatter intensity shown in Fig. 3a. The head generates 1024 dynamically focused beams and measures relative water depths over a 210° wide swath perpendicular to the vessel track. The MBES positioning was measured using a real-time kinematic GPS accurate to 0.01 m horizontally and 0.02 m vertically. The instrument has an internal inertial guidance system accurate to 0.02°. The survey was undertaken on 24/07/2024 on the vessel RV Macoma.

Four acoustic Doppler current profilers (ADCP’s) were deployed by the RV Macoma in the period September-August 2023 by the School of Ocean Sciences, Bangor University Staff. Due to operational constraints, the ADCP’s were deployed in pairs. The first two ADCP’s were Nortek Signature 500’s (ADCP 1, 2), and were deployed nearest the monopile, on 2024-04-27 06:00:00. The second deployment was of two Nortek signature 1000’s, at 55 m and 192 m from the east of the monopile. The ADCP at 55 m fell over shortly after deployment, and little useful data was collected. Thus, only ADCP 1,2, & 3 are shown.

### ADCP Instrument settings and post processing

All instruments were mounted on a small frame and were upward-looking. ADCP’s were set to record in beam coordinates, for 10-minute-long bursts, with an interval of 20 min, using all 5 beams at the highest resolution possible (Table 2). Post-processing of the ADCP data followed the procedure of^128^. TKE was calculated from the Reynolds Stresses using the 5-beam method. Noise floors for each instrument were calculated using the median value of TKE at slack water (defined as a burst having a depth mean current speed of less than 0.05 m s^−1^), and these noise floors were taken away from the values for TKE before multiplying by the density of seawater. As TKE measurements from field data are inherently noisy, a robust lowess filter (“rlowess” in MATLAB) was applied to the bed shear stresses derived from the TKE method with a 10-point window.

**Table 2:** ADCP deployment details

	ADCP1	ADCP2	ADCP3	ADCP4
Start time	23-Aug-2023 06:00:00	23-Aug-2023 06:00:00	30-Aug-2023 00:30:00	30-Aug-2023 00:30:00
End time	19-Sep-2023 07:38:31	19-Sep-2023 07:38:31	07-Sep-2023 13:08:31	07-Sep-2023 13:08:31
Location (Lat, long)	53.389133333, -3.68640500	53.389068333, -3.685691667	53.389018333, -3.685245000	53.388813333, -3.683120000
Distance from monopile (m)	23.33	24.97	55.05	192.20
Frequency (KHz)	500	500	1000	1000
Profile interval (s)	600	600	600	600
Burst interval (s)	1800	1800	1800	1800
Number of beams	5	5	5	5
Cell size (m)	0.5	0.5	0.2	0.2
Blanking distance (m)	0.5	0.5	0.1	0.1
Number of cells	60	60	149	149
Sample rate (Hz)	4	4	8	8
Successful deployment?	yes	yes	No, instrument rolled over on 30-Aug-2023 6:34:16	yes
Instrument noise floor (m s^−1^)	0.0192	0.0191	n/a	0.0227

Bed shear stress from the depth average variables was calculated via:

19 $${\tau }_{b}=\rho C{\bar{U}}^{2}$$

where the Chezy roughness is defined via:

20 $${C=\left(0.4/\log \left(h/{z}_{0}\right)-1\right)}^{2}$$

## Supplementary information

Supplementary information

Transparent Peer Review file

## Data Availability

Data generated used in the projection of the figures is available, alongside the model setup and laboratory data, via^129^: 10.5281/zenodo.15046910.
