# Supplementary material for: Turbulence drives seabed modification by offshore windfarms
Source: Nat Commun. 2026 Jun 3;17:7160. doi: 10.1038/s41467-026-73089-x (PMC13396209; doi:10.1038/s41467-026-73089-x)
Supplement: Supplementary file 1 — Supplementary information [file 41467_2026_73089_MOESM1_ESM.pdf]

Supplementary information for "Turbulence drives seabed modification by offshore windfarms". By:

Christopher A. Unsworth[1], Connor J. McCarron[2], Richard J.S Whitehouse[2], Thomas D.G. Benson[2], Ignacio Barranco[2&3], Mike Clare[4], James Waggitt[1], Lisa Skein[4], Veerle A.I. Huvenne[4], Martin J. Austin[1], and Katrien J.J. Van Landeghem[1].

[*]Corresponding author email address: christopher.unsworth@bangor.ac.uk
[1*]School of Ocean Sciences, Bangor University, Menai Bridge, Isle of Anglesey LL59 5AB, United Kingdom.
[2] Coasts and Oceans, HR Wallingford, Howbery Park, Wallingford OX10 8BA, United Kingdom.
[3] now at: Technology Centre for Offshore and Marine, Singapore (TCOMS). 2 Prince George's Pk, Singapore 118411
[4] National Oceanography Centre, Southampton, UK

Contents:

1) Comparison of model resolution with similar work
2) Definition of the models' sigma layers
3) Comparison of laboratory and modelled values for mean velocity and turbulent kinetic energy
4) Calculation of the areas of typical windfarm which could be affected by the results
5) References

1. Comparison of model resolution with other work

Upon building the mesh for the computation model, a comparison of monopile resolutions was looked at to find the optimal resolution. As different modelling studies has used a range of monopile sizes a normalised parameter was used, where the resolution was divided by the monopiles' circumference (Table S1). In our results a value of 0.0031 was found to be optimal for accurately modelling the flow around the monopile, a value similar to one used by a recent TELEMAC model[1]. We used a growth factor of 1.1 to grow the mesh from the monopile, ensuring high resolution close to the monopile to capture the shear layers.

Table S1. Comparison of the horizontal resolution of the monopile with other numerical models.

Source	Monopile diameter (mm)	Horizontal resolution of monopile in mm	Resolution / circumference
PhD. Christie, (2014).[2]	536	10 (π / 180)	0.0059
PhD. Yin, (2017).[3]	536	10 (π / 180)	0.0059
Research paper. Roulund *et al.*, (2005).[4]	536	27 (π / 63.5)	0.0160
PhD. Bourgoin, (2019).[1]	63	0.55 (π / 360)	0.0028
This study	250	2.45 (π / 320)	0.0031

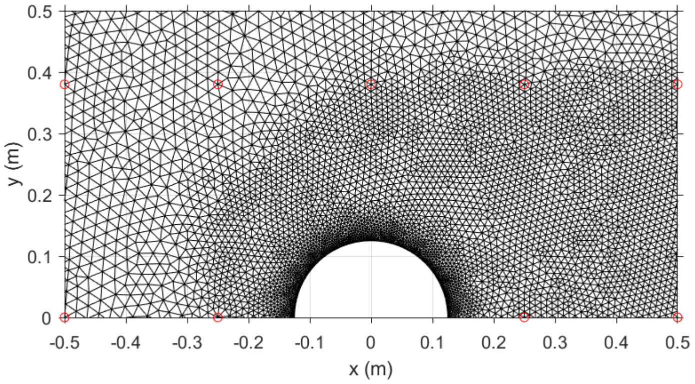

Figure S1. Example of the unstructured triangular mesh around the monopile. Red circles denote laboratory measurement locations which are fixed points in the numerical mesh. Flow direction is left to right.

A further 3 refinement regions were introduced to ensure the turbulent wake from the monopile was well resolved (Figure S2).

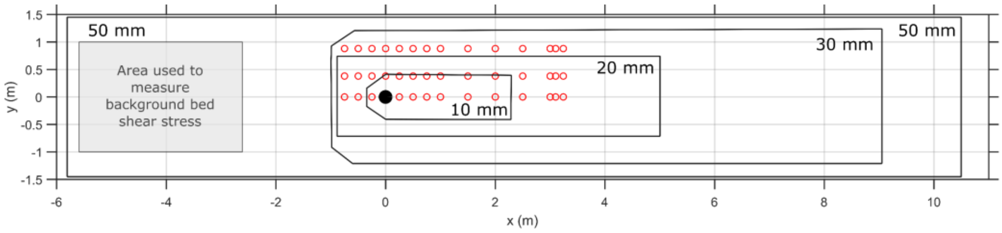

Figure S2. Horizontal mesh refinement regions, and laboratory measurement locations (red circles). Model boundary is the 50 mm box.

2. Definition of the models' sigma layers

TELEMAC3D uses a sigma style vertical layer system in which the 2D mesh is then extruded from the bed to the free surface. Here we increased resolution at the bed and free surface to properly resolve regions of high shear using the equation:

$$x_3^*(i_p) = \sigma_0 e^{k_a - \frac{(1-\sigma_0)k_a^2}{2k_m}} - \sigma_0$$

$$k_m = 2\frac{\ln 1 + 1/\sigma_0}{1 + \sigma_0}$$

$$k_a = k_m \frac{i_p - 1}{n_p - 1}$$

where i_p is the index of the plane under consideration (planes range from 1 to n_p, from the bottom to the free surface), vertical coordinate of each plane is x_3^*, σ_0 is a relaxation coefficient, in this case set to 0.02. With this number of planes and resolution, the bottom sigma layer had a y+ value >30, and the bottom 5 layers were within y+ < 300 to keep the near bed region well resolved and consistent with the roughness modelling approach. To ensure consistency at the top and bottom of the domain, minimum distances were set, 2 mm at the bottom and 3 mm at the surface, to ensure no collapsed or highly non orthogonal prisms around the monopile and rock armour. This profile is shown in Figure S3, with normalised values.

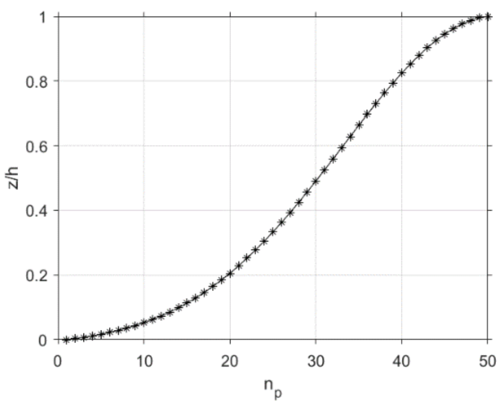

Figure S3. Non- dimensionalised vertical spacing of the sigma layers in the model.

3. Comparison of laboratory and modelled values for mean velocity and turbulent kinetic energy

The following figures (S4-S7) show the modelled values (lines) vs. the laboratory measurement (dots). Each laboratory measurement of velocity includes an error bar of 1 standard deviation from the mean.

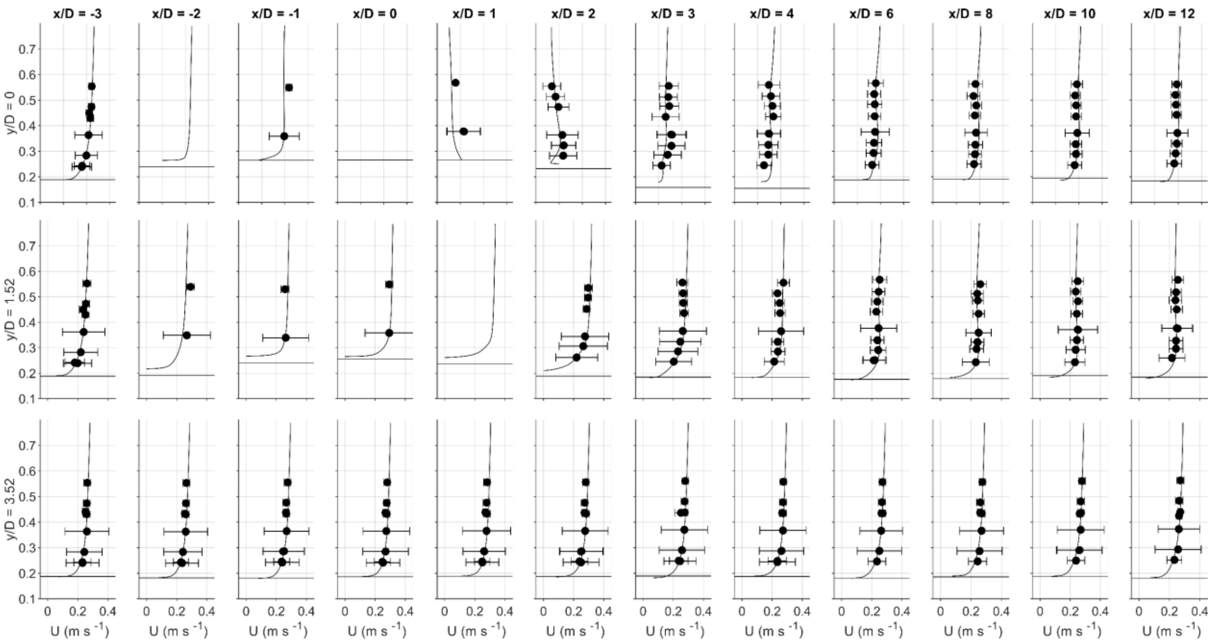

Figure S4. Comparison of mean U velocities from the labwork and numerical model. Mean U velocity from laboratory measurements (Black dots), with 1 standard deviation of plotted as whiskers. Model data is shown as a solid line. Lower laboratory datapoints contain more noise due to the Nortek Vectrino profiler requiring higher seeding levels than were possible in the laboratory.

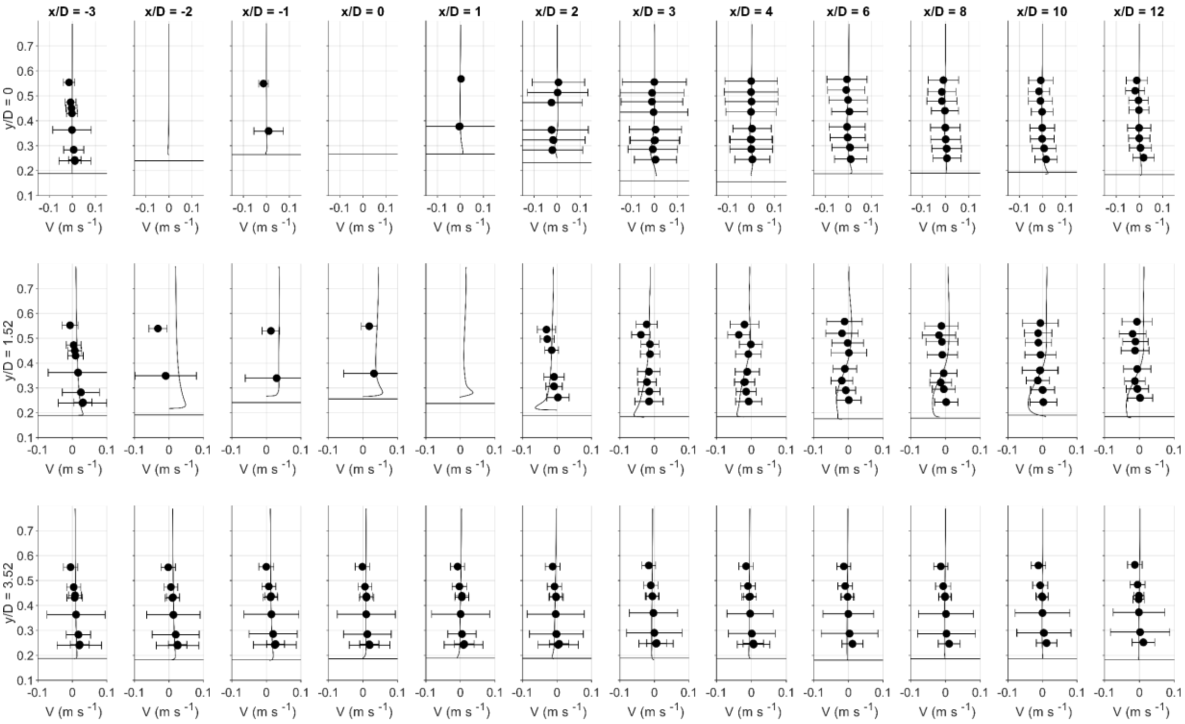

Figure S5. Comparison of mean V velocities from the labwork and numerical model. Mean V velocity from laboratory measurements (Black dots), with 1 standard deviation of plotted as whiskers. Model data is shown as a solid line. Noise levels in the laboratory data are higher overall relative to the magnitude of the velocity component, as the V velocity is quite small.

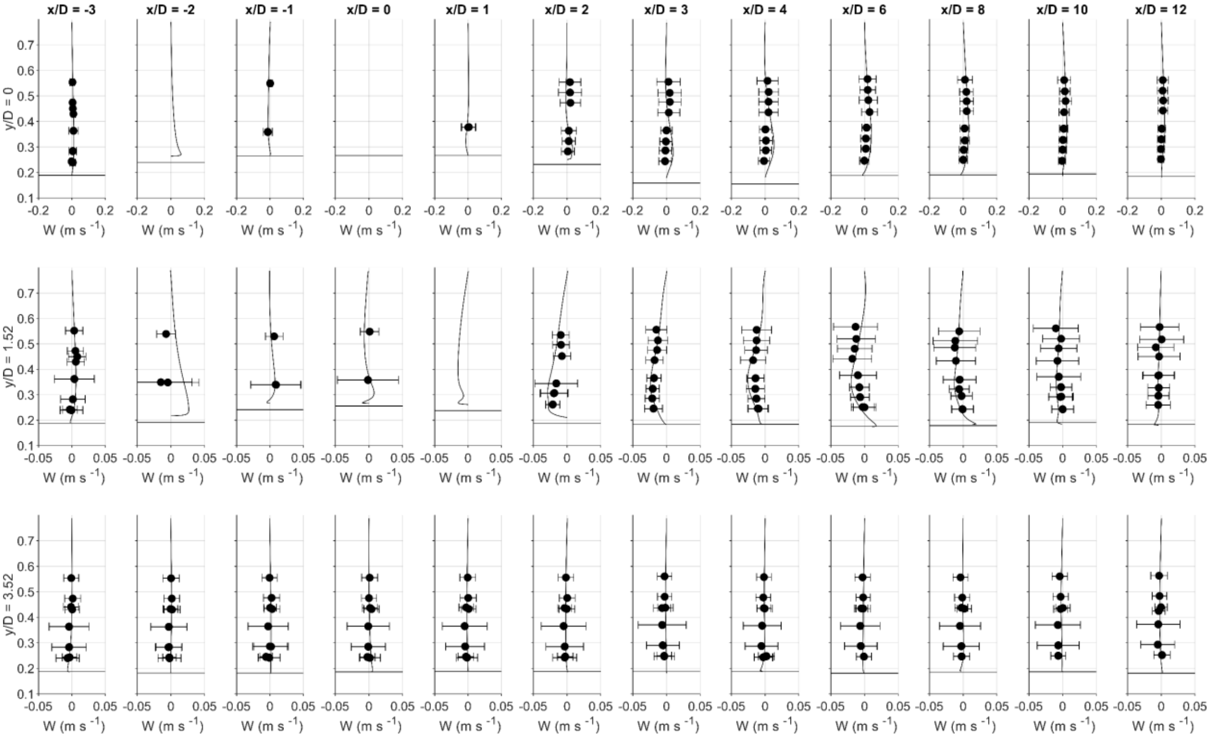

Figure S6. Comparison of mean W velocities from the labwork and numerical model. Mean W velocity from laboratory measurements (Black dots), with 1 standard deviation of plotted as whiskers. Model data is shown as a solid line. Noise levels in the laboratory data are higher overall relative to the magnitude of the velocity component, as the W velocity is quite small.

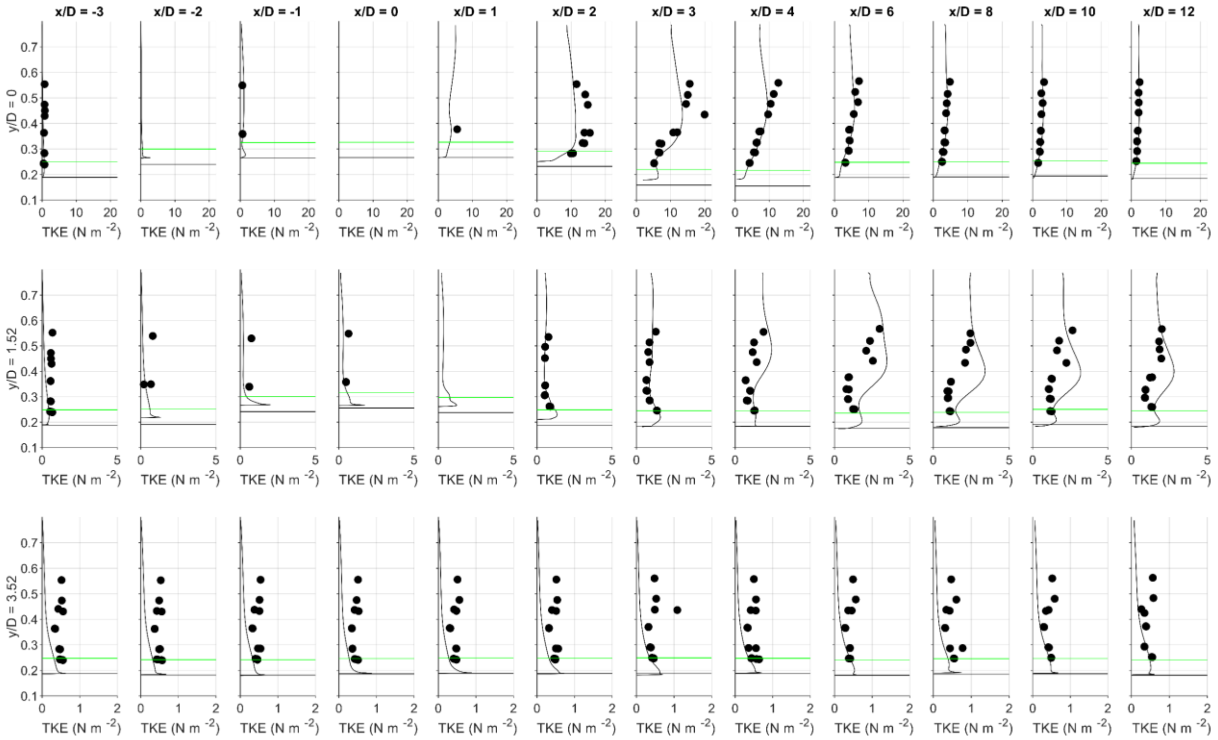

Figure S7. Comparison of turbulent kinetic energy (TKE) values from the labwork and numerical model. TKE from laboratory measurements (Black dots), and model data is shown as a solid line. As TKE implicitly incorporates noise, no error bars are shown. Green horizontal lines denote the 10% flow depth elevation where the Model data was extracted to estimate bed shear stress, for the lab data the nearest data point to that 10% elevation was used.

The numerical model was sampled at all of the locations in the laboratory where valid data was collected, so a direct comparison between laboratory and model could be calculated. A least squares linear regression was performed for each component of velocity and the Turbulent Kinetic Energy (TKE) (Table S2), which shows good agreement for the U velocity component, and acceptable agreement for the TKE. Across and Vertical velocity components (V and W) compare unfavourably, largely due to the velocity magnitudes being very similar in magnitude to the error of the measurements.

Table S2. Comparison of the horizontal resolution of the monopile with other numerical model's.

	Linear regression			
	slope	Intercept	R^2	RMSE
U	1.0375	-0.0058583	0.94	0.0174
V	1.1125	0.004419	0.382	0.0115
W	0.50403	-0.001555	0.0822	0.0135
TKE	0.79705	0.32837	0.858	1.03

As the measurements themselves have error, a reduced major axis regression was performed, which shows a better agreement for the V and W components (table S3). Mean absolute error, relative mean absolute error, Normalised standard error, Route mean squared error, and the Wilson score were also calculated (Table S3):

$$MAE = \frac{1}{N}\sum_{i=1}^{N}|y_{lab} - y_{mod}| \quad \text{S1}$$

$$RMAE = \frac{1}{N}\sum_{i=1}^{N}\left|\frac{y_{lab}-y_{mod}}{y_{lab}}\right| \quad \text{S2}$$

$$RMSE = \{[\sum_{i=1}^{N}(y_{lab} - y_{mod})]^2\}^{0.5} \quad \text{S3}$$

$$NSE = \frac{\sum_{i=1}^{N}(y_{lab}-\bar{y})^2 - \sum_{i=1}^{N}(y_{lab}-y_{mod})^2}{\sum_{i=1}^{N}(y_{lab}-\bar{y})^2} \quad \text{S4}$$

$$\mathrm{WS} = 1 - \left(\frac{(y_{lab}-y_{mod})^2}{\left((y_{lab}-\overline{y_{mod}})+(y_{lab}-\overline{y_{mod}})\right)^2}\right) \quad \text{S5}$$

Where, N = the number of observations, i = individual sample number, y_{lab}= laboratory measurement, y_{mod}= model measurement.

Table S3. Reduced major axis regression and statistical comparison.

	slope	intercept	MAE	RMAE	RMAE %	NSE	RMSE	WS
U	1.0700	-0.0130	0.0123	0.0002	0.0161	0.9287	0.0176	0.983
V	1.7953	0.0054	0.0075	0.0034	0.3352	17.112	0.0122	0.6618
W	1.7317	0.0002	0.0107	0.0048	0.4775	36.143	0.0140	0.4018
TKE	0.8605	0.2027	0.5584	0.0008	0.0762	0.000223	1.2086	0.9560

One possible source of systematic error can be the location error of the instrumentation in the laboratory. In order to test for the location error of the laboratory data, we followed the method of[5] and gradually increased the sampling window in the model to see if the MAE reduces. Figure 8 shows that increasing the spatial error window does not systemically reduce MAE, and therefore the location of the instrumentation data when sampling the model is appropriate.

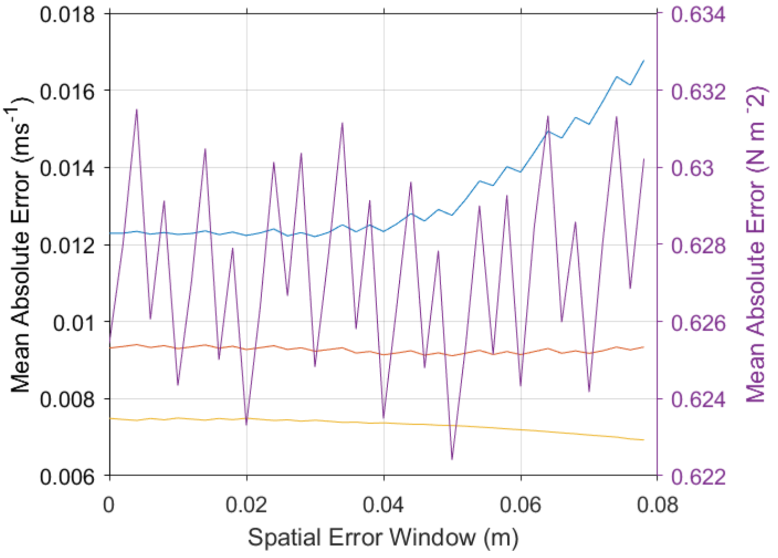

Figure S8. Testing Mean Absolute Error (MAE) variability due to instrument location error.

4. Calculation of the areas of typical windfarm which could be affected by the results

The table below outlines the calculations made to estimate the scale of windfarm infrastructure relative to a windfarms area "Infrastructure % Windfarm", but also the potential scale of the effect we demonstrate in the paper "Footprint % Windfarm". The monopile size and windfarm areas are based on typical windfarms of these scales (those built and planned) in the North West European Shelf.

Description	Symbol	Units	5 m monopile	15 m monopile
Wind Farm Polygon Area		km^2	9.67	78.00
		m^2	9,674,000	78,000,000
No. Monopiles	i	-	25	50
Monopile Diameter	M_{Di}	m	5.00	15.00
Monopile Radius	M_{Ri}	m	2.50	7.50
Individual Monopile Area	M_{Ai}	m^2	19.63	176.71
Rock Armour Radius Multiplier	n	-	3.30	5.30
Rock Armour Diameter	R_{Di}	m	16.50	79.50
Rock Armour Radius	$R_{Ri} = n*M_{Ri}$	m	8.25	39.75
Rock Armour + Monopile Area	$R_{Ai} + M_{Ai}$	m^2	213.82	4963.91
Rock Armour Area	R_{Ai}	m^2	194.19	4787.20
Total Monopile Area	M_A	m^2	490.87	8835.73
Total Rock Armour Area	R_A	m^2	4854.74	239359.91
Total Infrastructure Area	I_A	m^2	5345.62	248195.64
Infrastructure % Windfarm	$\%I_A$	%	0.06%	0.32%
Non-Dimensional Footprint Length	F_L^*	-	25	25
Non-Dimensional Footprint Width	F_W^*	-	6	6
Individual Footprint Length	F_{Li}	m	127.5	382.5
Individual Footprint Width	F_{Wi}	m	32.5	97.5
Individual Footprint Area	$F_{Ai} = \pi*F_{Li}*F_{Wi}$	m^2	13,018	117,162
Total Footprint Area	F_A	m^2	325,449	5,858,089
Footprint % Windfarm	$\%F_A$	%	3%	8%

NW European shelf area = 1,159,100 km^2

NW European offshore windfarm area (current and planned) = 44,155 km^2

% of the shelf potentially affected by the wakes of monopiles:

0.11% for 5 m monopile

0.3% for 15 m monopile

5. References.

1. Bourgoin, A. Modélisation de la turbulence engendrée par la morphologie dans le Raz Blanchard : approche régionale avec TELEMAC-LES. (Laboratoire universitaire des sciences appliquées de cherbourg, Caen, 2019).
2. Christie, E. K. Numerical modelling of morphological impacts of Offshore Wind Farms. (University of Liverpool, 2014).
3. Yin, Y. Turbulence model and Immersed Boundary method development in TELEMAC-3D for offshore structure modelling. (University of Liverpool, Liverpool, 2017).
4. Roulund, A., Sumer, B. M., Fredsøe, J. & Michelsen, J. Numerical and experimental investigation of flow and scour around a circular pile. *J. Fluid Mech.* **534**, 351–401 (2005).
5. Marjoribanks, T. I., Hardy, R. J., Lane, S. N. & Tancock, M. J. Patch-scale representation of vegetation within hydraulic models. *Earth Surf. Process. Landf.* **42**, 699–710 (2017).
