# Supplementary material for: Turbulence drives seabed modification by offshore windfarms
Source: Nat Commun. 2026 Jun 3;17:7160. doi: 10.1038/s41467-026-73089-x (PMC13396209; doi:10.1038/s41467-026-73089-x)
Supplement: Supplementary file 2 — Transparent Peer Review file [file 41467_2026_73089_MOESM2_ESM.pdf]

Peer Review File

Turbulence drives seabed modification by offshore windfarms

Corresponding Author: Dr Christopher Unsworth

This file contains all reviewer reports in order by version, followed by all author rebuttals in order by version.

Attachments originally included by the reviewers as part of their assessment can be found at the end of this file.

Parts of this Peer Review File have been redacted as indicated to remove third-party material.

Version 0:

Reviewer comments:

Reviewer #1

(Remarks to the Author)
This paper examines the potential impacts of wind turbine monopiles on the seabed using scaled physical experiments and 3D numerical simulations. Focusing on a monopile with rock armor at its base, the study evaluates the extent of sediment mobility driven by near-bed turbulence and wake effects. By incorporating turbulent kinetic energy (TKE) from both mean flow and wake-induced turbulence, the authors propose a method to estimate the affected area. Results reveal that sediment disturbance and bathymetric changes extend much farther downstream than previously predicted, with potential consequences for benthic habitats. The study underscores the importance of accounting for far-wake dynamics in environmental assessments of offshore wind farms.

This manuscript presents a compelling narrative that I found both interesting and enjoyable to read. The topic is highly relevant, particularly given the rapid expansion of offshore wind turbine deployment in various regions around the world. While technical, the study is sufficiently broad to appeal to the audience of Nature Communications, and the discussion aligns well with the journal's scope. The manuscript is well written, the figures are of good quality, and the methodologies are generally sound. Although this work may satisfy some of the criteria for publication in Nature Communications, I have a major reservation that currently prevents me from recommending acceptance.

My fundamental concern relates to the applicability and scalability of the results, which in turn affects the soundness of some of the broader discussions. The main point of this work is to demonstrate the extended spatial influence on bathymetry induced by the monopile of the turbine support structure, and thus the need for including the far-wake impacts in modeling efforts. This point is well taken, and I agree with the whole premise. However, the quantitative conclusions and the broader speculations drawn from them, are based on a single case study conducted under highly specific conditions. In my view, this limitation renders the discussion about implications at the shelf scale (lines 258–294) highly speculative. Below are four points that triggered my doubt:

1) Bedforms significantly affect hydraulic roughness, much more than skin drag. However, the numerical model employed in this study accounts only for grain roughness (i.e., skin drag) and does not include form drag. As bedforms develop, the local shear stress distribution changes, which could in turn affect the overall area of influence, i.e., the central result of this study. It is important to note that the authors do not model sediment transport (i.e., Exner equation), which may be a major limitation when considering the TKE discussion and the applicability of their conclusions. In this regard, the discrepancy between the observed extent of bathymetric change (i.e., the train of dunes) and the modeled area of increased TKE (lines 162–166) may point to a fundamental limitation of the model. I wonder whether the formation of the bedforms itself altered the local shear stress enough to halt their further development [1]. If so, the length of bedform development could be physical and not a limitation of the experiment run time. In this case then the results obtained via the TKE method may not be fully reliable, and the main quantitative conclusions would be less robust. Note that the TKE measurements and comparison are limited to 13.6D. It is unfortunate that the authors were unable to monitor the temporal evolution of the bedforms – using a moving sonar system for example. I believe that reaching equilibrium in bed evolution is an important prerequisite for drawing meaningful conclusions.

2) From literature (e.g., [2], [3]) —and as also acknowledged in this study— we know that the local scour around a cylinder

and the formation of bedforms are initiated by coherent flow structures shed by the obstacle (e.g., horseshoe vortices). These structures are strongly influenced by the Reynolds number (e.g., [4]). According to Table 1, the Reynolds number used in the experiment are approximately an order of magnitude lower than those from the field example used, and it’s reasonable to assume higher ranges of Reynolds number in the field. How does this discrepancy affect the results and thus how scalable are the observation of this specific case to the shelf-scale locations used by the authors in their discussion?

3) The choice of sediment size is justified to achieve similarity in the threshold of motion, which is a fair experimental assumption and premise. However, the ratio D/d (where D is the tower diameter and d is the grain size), also referred to as the relative coarseness in [5], has a direct impact on the scour magnitude. This was shown theoretically for both bridge pier [5] and hydrokinetic turbines [6] scour predictions. If the magnitude of the scour changes according to different D/d, it might as well affect the consequent bedforms generation and the actual extent of the overall influence. This adds to my doubt that the results presented may be case-specific and the scalability may not be straightforward.

4) In Table 1, the reported value of $\theta'_{SF/sub>}/\theta_{crit/sub>}$ exceeds 1 for the Field Example, indicating that sediment substrate is mobile and the conditions are not subcritical in the field. Unless I’m misinterpreting the table, this seems to contradict both the experimental setup and the authors’ claim in the introduction that field conditions are subcritical. While this inconsistency may not be a critical flaw since clear-water scour studies remain relevant, it does further question the representativeness of the results at the field scale, particularly given that the proposed numerical model assumes a fixed bed and does not simulate sediment transport.

These are critical points that the authors should address before this work can be considered for publication. I encourage the authors to perhaps incorporate additional analysis, where possible, or discussions to better support the scalability of their findings and strengthen the broader implications of the study. In parallel, clarifying the limitations of the modeling approach and resolving inconsistencies would enhance the manuscript’s impact. I look forward to the authors’ responses.

Additionally, I hope the following minor comments could help improve the quality and significance of the paper:
- There is a substantial body of research on the large-scale effects of marine energy technologies—such as hydrokinetic turbines and wave energy converters—on erodible bathymetry, sediment transport, and associated ecological impacts. Given the similarities in deployment environments and the nature of the direct impacts, it may be worthwhile to briefly review and reference this literature. This is a minor suggestion and ultimately a matter of the authors’ discretion.
- Line 28: missing “r” in the word “Through”.
- Lines 133 – 135: how was this (i.e., the length of mobile bed underestimation) assessed? is it shown anywhere?
- Line 137: unnecessary comma at the end of the line?
- Line 168 – 170: I think the development and evolution of the turbulent structures responsible for the local mobility of send in the presence of obstacles should be described better and upfront for the general reader to understand. This might imply restructuring the first two main subsections of the Results section and explain the turbulent processes before showing bathymetric and TKE results. Again, this might be ultimately a stylistic decision.
- Line 229 – 230: “bed shear stresses high enough to mobilize sediment under these “below threshold” conditions thus forming the bedforms”. I think this might be misleading because the conditions locally go indeed over the threshold. Maybe it can simply be reworded as: “The resultant three-dimensional wake flow generates near-bed turbulence that locally increase the shear stress over the threshold of motion thus forming the bedforms.”
- Figure 1c: I was surprised to see such a pronounced upwelling region in front of the monopile, as previous studies have shown that flow typically moves downward when encountering a cylinder, generating a recirculation zone and horseshoe vortices around the base. I assume this upwelling is the result of the specific upward-sloped design of the rock armor at the pier’s base—can you confirm if this is the case? I believe it would be worthwhile to expand the description of Figure 1c when discussing the flow development and turbulent structures around the monopile.
- Line 462: missing “/” between x and D.
- Line 504: something is missing at the end of the sentence.
- Line 520: I assume it should be “altitude” not “attitude”. Also, why was the accurate control of the instrument’s elevation not possible?
- Streamwise component of momentum equation (second question of Eq. 10): should be u and not i before $\partial u/\partial x$.

References
[1] J. Lee, A. Singh, and M. Guala, “On the Scaling and Growth Limit of Fluvial Dunes,” J. Geophys. Res. Earth Surf., vol. 128, no. 6, p. e2022JF006955, 2023, doi: 10.1029/2022JF006955.
[2] G. Kirkil, S. G. Constantinescu, and R. Ettema, “Coherent Structures in the Flow Field around a Circular Cylinder with Scour Hole,” J. Hydraul. Eng., vol. 134, no. 5, pp. 572–587, May 2008, doi: 10.1061/(ASCE)0733-9429(2008)134:5(572).
[3] C. Escauriaza and F. Sotiropoulos, “Initial stages of erosion and bed form development in a turbulent flow around a cylindrical pier,” J. Geophys. Res. Earth Surf., vol. 116, no. 3, pp. 1–24, 2011, doi: 10.1029/2010JF001749.
[4] C. Escauriaza and F. Sotiropoulos, Reynolds number effects on the coherent dynamics of the turbulent horseshoe vortex system, vol. 86, no. 2. 2011. doi: 10.1007/s10494-010-9315-y.
[5] C. Manes and M. Brocchini, “Local scour around structures and the phenomenology of turbulence,” J. Fluid Mech., vol. 779, pp. 309–324, 2015, doi: 10.1017/jfm.2015.389.
[6] M. Musa, M. Heisel, and M. Guala, “Predictive model for local scour downstream of hydrokinetic turbines in erodible channels,” Phys. Rev. Fluids, vol. 3, no. 2, p. 024606, 2018, doi: 10.1103/PhysRevFluids.3.024606.

Reviewer #2

(Remarks to the Author)
This paper presents the results of laboratory experiments and numerical simulations of the flow and bed disturbance around a protected wind turbine monopile. The experiments are original in that they provide novel data on the flow far downstream in the wake of the pile, alongside the presence of scour protection at the base of the pile. The numerical simulations are state of the art, based on the 3D version of OPENTELEMAC. The experimental data were used to validate the numerical simulations in the wake, in terms of both mean velocity and turbulent kinetic energy (TKE). The numerical results are then used to extrapolate the region of influence of the monopile in terms of bed mobility. The authors address three research questions: (1) What is the intensity and spatial scale of the hydrodynamic effects on the bed from a scour-protected monopile, accounting specifically for turbulence and not just mean flow? (2) What is the change in sediment transport capacity in the wider area away from the monopile when the seabed sediment is naturally immobile but close to the mobilisation threshold? (3) How might this consequently affect habitat composition and distribution, and its prediction? While the first question is addressed in the article, the second relies heavily on the model, for which additional details would be needed, and the third is very speculative, as no data are used to infer habitat modifications. I would have expected to see some numbers here! In my opinion, the technical part of the manuscript is interesting and could be published, provided additional information on and verification of the numerical simulations are provided. The third question is not mature enough at this stage and would require much more work, which I don't believe could be achieved through a major revision. Therefore, my recommendation would be to reject the manuscript, but encourage the authors to resubmit, potentially to a different journal.

Comments:

1) The predicted bed shear stress from this blended method shows the wake of the monopile to be dominated by TKE —> show the equation
2) Fig 1: You normalize y and x but not the elevation. Why ? Zoom in on the region around the pile to illustrate the flow around the structure (panel b : bed shear stress) how do you measure the bed shear stress ?
3) Which turbulence model is used in the simulations and how are the boundary conditions set, especially on the cylinder? What is the mesh resolution in the x, y and z directions around the cylinder and near the bed? I found the answers in the supplementary material, but it is disconcerting to see the results without knowing the underlying modelling assumptions. TELEMAC3D is not known to work very well for simulating flow around cylinders, at least in the near field. It is important to provide many more comparisons of the flow in the near and far fields in order to support the use of the model for extrapolating measurements.
4) l. 349-356: This is highly qualitative and speculative. Can you quantify these arguments, for example by giving typical values of the turbidity levels in the wake and comparing them with those outside the wind farm?
5) An important issue with this work is that all these quantities are obtained from parameterisation in the numerical simulation, and there is no experimental data to support the estimation of bed shear stress based on hydrodynamics, except for bed elevation.
6) Table 1: Table 1: Why use the same particles in a scaled model? Sediment dynamics will differ significantly; should the author not use a Shields similarity to match bed mobility?
7) The measurements do not cover the 25D extension of the wake therefore the conclusion of the article is an extrapolation based on the simulation. This result cannot be fully trusted until the simulation has been validated more carefully.
8) How can the wake be accurately represented if the no-slip boundary condition on the monopile is not accounted for? How are the BCs for turbulence specified? How is vortex shedding predicted? The authors need to present results on wake dynamics from the simulations, such as the Strouhal number, as well as some snapshots of coherent vortices shed from the cylinder.
9) A domain covering a sandy section of the flume experiments (3 m x 16.2 m) was created with a resolution of 50 mm (see Figure 4d). This is relatively large compared to the thickness of the boundary layer (BL) around the monopile.
10) « Flow depth and discharge were specified at the inlet, with a power law profile specified in the vertical. The flow depth and discharge at the inlet was fixed at 0.6 m and 0.435 m3 s-

1, respectively. The outlet was given a fixed discharge of -0.435 m3 s-1, and a free depth to account for the water surface slope »: The way the boundary conditions are set here doesn't make sense if the flow is subcritical. Please explain.

Typo: l.571: missing 0: 1^... --> 10^...

Reviewer #3

(Remarks to the Author)
Unsworth et al. present a fascinating manuscript on how offshore wind turbines can substantially alter the seabed. As my expertise is in marine ecology (and not physical oceanography), I focus my review on the ecological aspects and implications from the manuscript.

Overall: The authors' finding that the seabed sediment is altered up to 25x the monopile foundation diameter downstream is a novel finding that has the potential to change how we think about potential environmental. impacts of offshore wind energy. Additionally, the manuscript is particularly timely as, especially in the U.S., there has been a high degree of uncertainty regarding the level of impacts from offshore wind energy development. Unsworth et al. helps resolve some of this scientific uncertainty. I suggest that the manuscript be considered for acceptance to Nature Communications following a minor revision.

I've included several comments that the authors wish to consider addressing:

While I realize that the study focused on a single turbine, I suggest that the authors consider a bit more about what may happen with multiple turbines in the same wind farm. The calculations in Appendix 1 that were also verbally stated in the results/discussion of what to expect with a given number of turbines were really helpful. However, in the U.S., monopile foundation turbines are typically spaced about 1 nmi apart (I'm not sure how this differs in Europe and Asia), but I am keen to understand how the turbulence fields associated with multiple individual wind turbines may interact with one another. In particular, would the authors suggest that the magnitude of impacts may increase through interactions of turbulence and mean flow from nearby wind turbines, including those upstream, downstream, or positioned laterally? This would likely be a hypothetical statement, but I think mentioning the potential for interactive synergies could be worthwhile, especially from an ecological perspective. This is because wind turbines present such a unique configuration of artificial structure in the ocean given that hundreds of turbines are often installed in proximity to one another within a given wind farm.

This may also be hypothetical, but I'm curious – do the authors think that similar processes may occur around floating turbines? It could be interesting to mention this as a future research direction, especially in deeper waters where floating turbines are being considered (or are already being installed!).

Marine spatial planning - Marine spatial planning is mentioned in the abstract and in a few other
locations. If space permits, I would encourage the authors to describe their vision more fully for how their findings should be (or could be) incorporated into marine spatial planning frameworks. The study findings could be quite catalytic for marine spatial planning, and for the calculation/estimation of cumulative impacts.

Scour protection design – I see potential application of the study findings to help industry/developers better design scour protection. Are there general recommendations that you would have for developers on how to best design scour protection to minimize turbulence (if that is the goal…)?

Line 60 – Consider clarifying the 80%. Is that 80% of turbines globally that are monpiles? And, did the citation for the 80% value appear earlier in the sentence?

Line 66-68 – Suggest clarifying this sentence. I had trouble following it.

Line 94-95 – Is there a particular threshold for "deep" here? I ask this because floating turbines are being considered in deep waters instead of monopiles, in some cases (and many cases in the U.S.).

Line 361-362 – The sentence on artificial reefs is a bit misleading. I suggest clarifying. Artificial reefs are typically considered to have a mixture of unintended ecological consequences and – in some cases – ecological benefits.

Thank you for the opportunity to review your manuscript!

-Avery Paxton

Version 1:

Reviewer comments:

Reviewer #1

(Remarks to the Author)
Dear authors,

I have reviewed the revised manuscript together with the authors' responses to my comments on the original submission. The authors have responded satisfactorily to most of my comments, and I believe this has resulted in a substantially improved manuscript. I particularly appreciate the addition of the new field data, which further reinforces the authors' central argument that wake turbulence (measured while accounting for wake-added TKE) is the main driver of bathymetric impacts that develop over much longer distances than typically considered.

As a result, my original concerns regarding the specificity of the results have been partially addressed, and I believe the revised manuscript now presents much stronger and more compelling arguments. I still believe—and note that this concern was also shared by Reviewer #2—that many of the arguments and conclusions related to potential ecological implications at the shelf scale remain somewhat speculative. However, I also consider this acceptable, as these discussions naturally stem from the core results of the work (i.e., the extended bathymetric effects observed both experimentally and numerically). These are framed as potential consequences, and I am therefore comfortable with this level of speculation.

That said, a few comments remain:

1) I do not fully understand the reply to my comment #1. The authors state that "the resolution of the model is high enough to resolve the ripples and implicitly create the form drag which you mention." Are the authors using the laser scan from the experiment as a DEM for the numerical simulations? If so, I may have missed this detail in the methodology, and I suggest clarifying it.

2) I disagree with the authors' response to my comment #3. I reviewed Section 7.2.6 of Scour at Marine Structures (Whitehouse, 1998), which is cited by the authors, and I understand the premise of their argument. However, this interpretation is in direct disagreement with the data presented in Figure 4 of (Manes and Brocchini 2015, please see figure in the attachment), which clearly shows that dimensionless scour depth varies for values of the ratio D/d_{50} well beyond 50.
The data combines several (more recent) studies: (Ettema 1980; Sheppard et al. 2004; Ettema et al. 2006; Lança et al. 2013).

While I agree that scour is primarily controlled by flow intensity and that scaling based on the threshold of motion is appropriate, I also believe that other parameters—such as relative coarseness—play an important role. This is physically intuitive, and recent literature supports this view. Even if the effects on bedform generation might be minor, I believe the statement in the authors' response that "the local scour will [not] be controlled by […] relative coarseness" is conceptually incorrect.

I therefore encourage the authors to further review the relevant literature (perhaps starting with Manes & Brocchini, 2015) and to expand or refine their discussion accordingly. These issues directly relate to my original concerns regarding the scalability of the results. While I appreciate the revised wording used by the authors when applying their findings to the field scale (e.g., "Using our laboratory results as a guide rather than the field evidence…"), I still believe the authors should explicitly discuss scalability in terms of the main physical parameters (e.g., grain size distribution, relative coarseness, Reynolds number, etc.). In this regard: what uncertainty is associated with the predicted affected shelf areas (km2) of their real world examples? I suggest that these aspects be discussed either in the methodology section or, at minimum, in the Supplementary Information.

3) I understand the authors' intention to keep the manuscript focused on wind energy installations, and I acknowledge that this is a reasonable choice. However, statements in the article such as "While scour adjacent to infrastructure has been studied extensively, the impact on sedimented seafloors further downstream has been overlooked, despite its importance for environmental risk assessment" are not accurate. Given that the scour process and its associated downstream impacts on sedimented seafloors are phenomenologically similar, it is misleading to claim that such downstream impacts have been overlooked (at least by the scientific community). This statement risks disregarding a substantial body of prior work (some examples: (Neill et al. 2009; 2012; Fairley et al. 2015) and more for tidal environments, or (Musa et al. 2018) for river environments). I suggest either acknowledging this existing literature or revising the statement accordingly.

4) Lines 317-326: It may be difficult for readers to understand the distinction between "non-wake" and "wake" conditions (which depend on tidal direction) based on the current text and Figure 4 alone. I suggest briefly explaining this distinction here to improve the transition at line 317.

Additionally, the general readership of Nature Communications may not be familiar with bedform regimes and transitions (Figure 4 and the associated discussion). It may therefore be beneficial to provide additional explanation, unless this is already addressed elsewhere in the manuscript (in which case I apologize for overlooking it).

In conclusion, I would like to emphasize that none of the points raised above constitute major flaws, and I continue to believe that the study has merit and is suitable for publication in Nature Communications.

References
- Ettema, R. 1980. “Scour at Bridge Piers.” Department of Civil Engineering, University of Auckland.
- Ettema, Robert, Gokhan Kirkil, and Marian Muste. 2006. “Similitude of Large-Scale Turbulence in Experiments on Local Scour at Cylinders.” Journal of Hydraulic Engineering 132 (1): 33–40. https://doi.org/10.1061/(ASCE)0733-9429(2006)132:1(33).
- Fairley, I., Ian Masters, and H. Karunarathna. 2015. “The Cumulative Impact of Tidal Stream Turbine Arrays on Sediment Transport in the Pentland Firth.” Renewable Energy 80: 755–69. https://doi.org/10.1016/j.renene.2015.03.004.
- Lança, Rui M., Cristina S. Fael, Rodrigo J. Maia, João P. Pêgo, and António H. Cardoso. 2013. “Clear-Water Scour at Comparatively Large Cylindrical Piers.” Journal of Hydraulic Engineering 139 (11): 1117–25. https://doi.org/10.1061/(ASCE)HY.1943-7900.0000788.
- Manes, Costantino, and Maurizio Brocchini. 2015. “Local Scour around Structures and the Phenomenology of Turbulence.” Journal of Fluid Mechanics 779: 309–24. https://doi.org/10.1017/jfm.2015.389.
- Musa, Mirko, Craig Hill, Fotis Sotiropoulos, and Michele Guala. 2018. “Performance and Resilience of Hydrokinetic Turbine Arrays under Large Migrating Fluvial Bedforms.” Nature Energy 3 (10): 839–46. https://doi.org/10.1038/s41560-018-0218-9.
- Neill, S. P., James R. Jordan, and Scott J. Couch. 2012. “Impact of Tidal Energy Converter (TEC) Arrays on the Dynamics of Headland Sand Banks.” Renewable Energy 37 (1): 387–97. https://doi.org/10.1016/j.renene.2011.07.003.
- Neill, S. P., Emmer J. Litt, Scott J. Couch, and Alan G. Davies. 2009. “The Impact of Tidal Stream Turbines on Large-Scale Sediment Dynamics.” Renewable Energy 34 (12): 2803–12. https://doi.org/10.1016/j.renene.2009.06.015.
- Sheppard, D. Max, Mufeed Odeh, and Tom Glasser. 2004. “Large Scale Clear-Water Local Pier Scour Experiments.” Journal of Hydraulic Engineering 130 (10): 957–63. https://doi.org/10.1061/(ASCE)0733-9429(2004)130:10(957).

Reviewer #2

(Remarks to the Author)
Reviewer #2 (Remarks to the Author):

This is my second review of the manuscript. The new section on field observations addresses my previous concern that the main conclusions were not supported by sufficient evidence. However, as you will see below, there are still a number of points that require attention to ensure reproducibility of the work. These include details about the boundary conditions and roughness modelling, the near-wall resolution in dimensionless units, and the characterisation of the bedforms in the experiments. While the author's response regarding the Strouhal number is satisfactory, this new result should also be included in the manuscript for the benefit of future readers. In conclusion, I am now ready to accept the manuscript, provided additional information is included in the main text or supplementary materials.

--
This paper presents the results of laboratory experiments and numerical simulations of the flow and bed disturbance around a protected wind turbine monopile. The experiments are original in that they provide novel data on the flow far downstream in the wake of the pile, alongside the presence of scour protection at the base of the pile. The numerical simulations are state of the art, based on the 3D version of OPENTELEMAC. The experimental data were used to validate the numerical simulations in the wake, in terms of both mean velocity and turbulent kinetic energy (TKE). The numerical results are then used to extrapolate the region of influence of the monopile in terms of bed mobility. The authors address three research questions: (1) What is the intensity and spatial scale of the hydrodynamic effects on the bed from a scour-protected monopile, accounting specifically for turbulence and not just mean flow? (2) What is the change in sediment transport capacity in the wider area away from the monopile when the seabed sediment is naturally immobile but close to the mobilisation threshold? (3) How might this consequently affect habitat composition and distribution, and its prediction? While the first question is addressed in the article, the second relies heavily on the model, for which additional details would be needed, and the third is very speculative, as no data are used to infer habitat modifications. I would have expected to see some numbers here! In my opinion, the technical part of the manuscript is interesting and could be published, provided additional information on and verification of the numerical simulations are provided. The third question is not mature enough at this stage and would require much more work, which I don't believe could be achieved through a major revision. Therefore, my recommendation would be to reject the manuscript, but encourage the authors to resubmit, potentially to a different journal.
Reply: We are glad you find the technical aspects of the work interesting and hope you will find the added details to the modelling satisfactory. After reflecting on the editors and reviewers' comments we have included recent field data into the revised manuscript which demonstrates the turbulent wake effect of increasing bed shear stress at an operational offshore wind farm. We believe this new field evidence adds validity to our new method for real world conditions.
Whilst we cannot assess the exact impact that these turbulent wakes are having for every offshore windfarm in the world, we can at least present the potential scale of the impact – which is what we have tried to do with our shelf-scale estimation. We do not attempt to say that these scales are exactly what will happen – but that this is a process which needs to be taken into account when designing this renewable infrastructure. For the impacts to marine life – we have removed this as one of our aims, as any habitats modelling and quantification of the effect in the laboratory experiment would be hypothetical, and at our field site specific – as such we have changed aim three to “how might accelerated seabed mobility affect seabed composition when the bed is already mobile?” which is our new field case (Figure 3a). We have attempted to reinforce the linkages between physical environment and habitats. Variables like temperate, depth and, of importance in this paper, sediment grain size and bed shear stress are key variables in the EUNIS classification scheme. As you say, adding in a section of work showing these changes for our field site is essentially another paper entirely.
We hope you find our revised, and focused revision acceptable

---
Thank you for your response and for substantially revising the manuscript. I believe that adding the field data is a very good and convincing choice. I am also happy for the impact on seabed habitats and biotic interactions to remain a discussion point.

Comments:

1) The predicted bed shear stress from this blended method shows the wake of the monopile to be dominated by TKE —> show the equation
--
Reply: This has been included as Equation 11 in the methods section
---
Good, thank you for the addition.

2) Fig 1: You normalize y and x but not the elevation. Why ? Zoom in on the region around the pile to illustrate the flow around the structure (panel b : bed shear stress) how do you measure the bed shear stress ?
--
Reply: The z elevation was not normalised as a stylistic choice. The point of the figure is not to show the flow structure at the monopile in a lot of detail – this has been published many times before - but to focus on the turbulent wake. The method of measuring bed shear stress in the laboratory, and from the numerical model is described in the method section, but briefly, we used a Nortek Vectrino profiler in the laboratory to measure the near bed mean flow and turbulence, and we calculated the bed shear stress via the method of Soulsby, wherein the TKE at 10% of the flow depth was multiplied by a constant (0.19) to produce a bed shear stress.

I won't insist on the depth normalization. I do believe that the bead shear stress data upstream the pile would be interesting and they could have been added to the supplementary file « Model Validation ».

3) Which turbulence model is used in the simulations and how are the boundary conditions set, especially on the cylinder? What is the mesh resolution in the x, y and z directions around the cylinder and near the bed? I found the answers in the supplementary material, but it is disconcerting to see the results without knowing the underlying modelling assumptions. TELEMAC3D is not known to work very well for simulating flow around cylinders, at least in the near field. It is important to provide many more comparisons of the flow in the near and far fields in order to support the use of the model for extrapolating measurements.
--
Reply: We do understand your frustration, the paper is ordered this way to conform to the Nature publication format standard. TELEMAC-3D has been shown to work well around cylinders using the turbulence model we have used (Spalart and Allmaras 1992), with high enough resolution at the monopile. Following (Bourgoin 2019; Bourgoin et al. 2021; 2020), we used a monopile resolution of 2.4 mm. The lowest sigma layer is 2 mm above the bed.
We hope the comparison of laboratory measured and numerical modelled bed shear stresses shown in Figure 8 is convincing. There is also a comparison of the profiles of U,V,W and TKE in the supplementary section.

I understand that the format of the Nature paper does not allow the details of the model to be introduced in the main text. However, to make the work fully reproducible, it is important to clarify how the boundary conditions for nuTilda are specified for the smooth cylinder wall and rough bed. One important point that is not mentioned and which I have been unable to find is how bed roughness is taken into account in the TKE. How is the TKE calculated in the Spallart–Almaras model? This could be added for completeness and reproducibility. Also, just to clarify, you do not account for the bedforms in the simulation? You use a constant bed roughness everywhere? Did you use the bed scan to build the bathymetry in the model? The bed

roughness might play a role in the bed shear stress estimate so it seems important to clarify this point.

4) l. 349-356: This is highly qualitative and speculative. Can you quantify these arguments, for example by giving typical values of the turbidity levels in the wake and comparing them with those outside the wind farm?
--
Reply: The purpose of this paragraph is to discuss the implications of offshore wind farms on seabird foraging strategies. The papers cited all have different measurements of the environmental conditions (e.g. Secchi Depth); there is not one universal method as this is a recent and evolving subject. We have changed to language in this paragraph to make sure we are not saying that our manuscript is showing changes in suspended sediment concentration. Our work sits alongside others in trying to highlight the range of effects on seabird foraging strategies that offshore windfarms can have - i.e. it is not just purely a negative effect for all seabirds and we believe the environmental impact assessments of offshore windfarms needs to take a more holistic view
---
As I mentioned earlier, I am happy with the new wording of this section. It is an interesting topic that merits a full paper (or many) in its own right.

5) An important issue with this work is that all these quantities are obtained from parameterisation in the numerical simulation, and there is no experimental data to support the estimation of bed shear stress based on hydrodynamics, except for bed elevation.
--
Reply: We believe the reviewer is incorrect with this statement. We report extensive largescale laboratory measurements of bed shear stress that have been obtained using robust and rigorous methodologies and these provide the foundation upon which the numerical model is developed; they are compared with the numerical model in Figure 8. Unless a numerical model is a direct numerical simulation of all of the scales of turbulence, then it will have parameterisation – this is the vast majority of numerical models used in environmental sciences and they are well supported by validation against observation
---
Let me phrase this differently. You have not explained precisely how you processed the data to obtain the bed shear stress using the mean flow method. How do you estimate bed roughness? How much is present at each point? What precise expressions do you use? You also claim that other methods, such as the Reynolds stress method, are inaccurate by ±10%. The TKE method is also quite indirect. It is important to provide more details on this crucial point to make the analysis fully reproducible. You could provide a comparison in the supplementary material for example. It would also be important, to be fair, to introduce error bars on the laboratory-measured bed shear stress. On another note, did you determine the wavelength and amplitude of the bedforms along the two transects shown in the insets of Figure 1(a)? If so, that information would be useful to provide.

6) Table 1: Table 1: Why use the same particles in a scaled model? Sediment dynamics will differ significantly; should the author not use a Shields similarity to match bed mobility?
--
Reply: Good point, I cannot remember why we used a field example at 60 cm/s. This table has been updated with representative flow conditions.

OK.

7) The measurements do not cover the 25D extension of the wake therefore the conclusion of the article is an extrapolation based on the simulation. This result cannot be fully trusted until the simulation has been validated more carefully.
--
Reply: We believe the validation of the model is excellent as demonstrated by figure 8 and the model validation supplementary section. If we cannot use model data outside of the validated locations, then not a single meteorological or oceanographic model would be valid. Given the validation shown in Figure 8 in the manuscript and in the supplementary material, we are confident that the model is representing the processes very well.

Although it is not quantitative, the use of field data provides strong support for the significantly larger extent of the increased bed shear stress wake region. This is a compelling argument, and I am satisfied with the response.

8) How can the wake be accurately represented if the no-slip boundary condition on the monopile is not accounted for? How are the BCs for turbulence specified? How is vortex shedding predicted? The authors need to present results on wake dynamics from the simulations, such as the Strouhal number, as well as some snapshots of coherent vortices shed from the cylinder.
--
Reply: The monopile uses a no-slip condition at the monopile, with zero roughness. This has now been clarified in the methods section. The turbulent wake in the model had a dominant eddy shedding frequency of 0.166 hz, giving a Strouhal number of 0.172, which compares very well with the prediction
---
In line with comment 4 above, I still think more information is needed on the flow and turbulent boundary conditions (including the effect of roughness), as well as an estimation of the initial grid size at the cylinder in wall units. This will help to confirm that the boundary layers are well resolved. Grid size in SI units is not helpful here. I have seen the FFT plot in the wake and the Strouhal number estimate in response to Reviewer #1. I believe that some of this information should be included in the manuscript. As your work focuses on the wake, including these quantities is essential to strengthening your results. Snapshots of the Q criterion would also be useful, but I don't want to force you to include them.

9) A domain covering a sandy section of the flume experiments (3 m x 16.2 m) was created with a resolution of 50 mm (see Figure 4d). This is relatively large compared to the thickness of the boundary layer (BL) around the monopile.
--
Reply: As per the methods section, the resolution of the horizontal mesh at the boundary of the model is 50 mm, but there are multiple regions of higher resolution within this to properly resolve the turbulent wake, see figure below:
[…]
Vertical spacing of the sigma layers in the model, with normalised vertical coordinates (z elevation / depth) and sigma layer numbering from zero (the bed) to the surface (50).

As with comment 8, I would like estimates of the cylinder grid size in wall units (minimum, maximum and mean, for example) to support good boundary layer resolution, or at least to provide quantitative information for reproducing these results with TELEMAC or another CFD code.

10) « Flow depth and discharge were specified at the inlet, with a power law profile specified in the vertical. The flow depth and discharge at the inlet was fixed at 0.6 m and 0.435 m3 s-1, respectively. The outlet was given a fixed discharge of -0.435 m3 s-1, and a free depth to account for the water surface slope »: The way the boundary conditions are set here doesn't make sense if the flow is subcritical. Please explain.
--
Reply: For each iteration the free surface is first solved using the wave equation, then the velocities are solved. The velocity at the boundary will then be scaled to give the prescribed outflow discharge whilst maintaining the cross-sectional shape of the flow field.

I still don't understand this set of boundary conditions. To me, a natural choice would be: an imposed discharge upsream and an imposed flow height upstream. The predicted slope of the free surface would represent the required pressure gradient to sustain the imposed discharge. If bedforms are generated and evolve over time, the friction will increase and so will the slope of the free surface from upstream to downstream. But it should be slow compared with the hydrodynamic time-scale.

Reviewer #3

(Remarks to the Author)
Unsworth et al. have revised their manuscript to thoroughly address my suggestions and comments, including those on scour protection, decommissioning, and marine spatial planning. In response to other reviewers' suggestions, Unsworth et al. have further strengthened the manuscript by including field data from two wind farms that help verify the real-world application of their model and laboratory experiments. As such, I recommend that the manuscript be considered for acceptance to Nature Communications.
-Avery Paxton

Version 2:

Reviewer comments:

Reviewer #1

(Remarks to the Author)
The authors have addressed my comments and suggestions raised during the review process. The revisions have improved the clarity and impact of the manuscript and have adequately resolved the points previously identified. I therefore believe the revised manuscript can now be considered for acceptance in Nature Communications.

Reviewer #2

(Remarks to the Author)

The authors replied satisfactorily to my comments and questions. I am glad to accept the manuscript.

Open Access This Peer Review File is licensed under a Creative Commons Attribution 4.0 International License, which permits use, sharing, adaptation, distribution and reproduction in any medium or format, as long as you give appropriate credit to the original author(s) and the source, provide a link to the Creative Commons license, and indicate if changes were made.
In cases where reviewers are anonymous, credit should be given to 'Anonymous Referee' and the source.
The images or other third party material in this Peer Review File are included in the article's Creative Commons license, unless indicated otherwise in a credit line to the material. If material is not included in the article's Creative Commons license and your intended use is not permitted by statutory regulation or exceeds the permitted use, you will need to obtain permission directly from the copyright holder.
To view a copy of this license, visit https://creativecommons.org/licenses/by/4.0/

Response to Reviews:

Reviewer #1 (Remarks to the Author):

This paper examines the potential impacts of wind turbine monopiles on the seabed using scaled physical experiments and 3D numerical simulations. Focusing on a monopile with rock armor at its base, the study evaluates the extent of sediment mobility driven by near-bed turbulence and wake effects. By incorporating turbulent kinetic energy (TKE) from both mean flow and wake-induced turbulence, the authors propose a method to estimate the affected area. Results reveal that sediment disturbance and bathymetric changes extend much farther downstream than previously predicted, with potential consequences for benthic habitats. The study underscores the importance of accounting for far-wake dynamics in environmental assessments of offshore wind farms.
This manuscript presents a compelling narrative that I found both interesting and enjoyable to read. The topic is highly relevant, particularly given the rapid expansion of offshore wind turbine deployment in various regions around the world. While technical, the study is sufficiently broad to appeal to the audience of Nature Communications, and the discussion aligns well with the journal's scope. The manuscript is well written, the figures are of good quality, and the methodologies are generally sound. Although this work may satisfy some of the criteria for publication in Nature Communications, I have a major reservation that currently prevents me from recommending acceptance.
My fundamental concern relates to the applicability and scalability of the results, which in turn affects the soundness of some of the broader discussions. The main point of this work is to demonstrate the extended spatial influence on bathymetry induced by the monopile of the turbine support structure, and thus the need for including the far-wake impacts in modeling efforts. This point is well taken, and I agree with the whole premise. However, the quantitative conclusions and the broader speculations drawn from them, are based on a single case study conducted under highly specific conditions. In my view, this limitation renders the discussion about implications at the shelf scale (lines 258–294) highly speculative. Below are four points that triggered my doubt:

Reply: We appreciate the kind comments here and are glad you accept the overall premise of our work. Your point about the specificity of the results which we have presented is also well taken. In our revised manuscript, we have sought to address these concerns by demonstrating that turbulence (measured via turbulent kinetic energy) is the main driver of sediment transport near offshore wind farms by using a combination of recent bathymetry surveys (new Figure 3) and acoustic Doppler current profiler (ADCP) data (new Figures 4-5). These new data cover over a month of tides and are likely to be the nearest bottom-mounted ADCP data of any offshore monopile collected to date (20 – 25 m from monopile, which was 4.7 m in diameter). We found that TKE as measured by the ADCP over this time period can also explain the alteration of the seabed in the turbulent wake of the monopile.

We have used the same methods of calculating bed shear stress as we used in our numerical model – both a 2D depth averaged method and using the TKE at 10% of the flow depth (the "TKE method"). When the ADCP's were measuring "normal" flow conditions (i.e. unaffected by turbulent wakes from the monopiles) these two methods produced very similar results – as would be expected for well-developed open channel flow (Nezu and Nakagawa 1993). However, when they were measuring the turbulent wake emanating from the monopile the bed shear stress produced via the TKE method was substantially higher. We then estimated the transport stage for the sandy sediment at this field site, and the implication of using the TKE method becomes clear – it predicts conditions during spring tides in the turbulent wake which are above dune forming conditions; whilst the 2D depth

averaged method prediction was still within the bedform forming regime. These data cover flow depths between 12 and 21 m, with mean velocities ranging up to 0.9 m/s.

We appreciate the concern around how we have tried to calculate the implication of our work at the self-scale. We do feel it is necessary to perform this analysis as it helps the reader understand the potential scale of the effect – but we do keep it as a *potential* effect and are clear that measurements and appropriate modelling are needed to know what the exact effects will be for each windfarm or monopile. We hope our work will highlight this need to regulators, windfarm developers and the consultants which undertake it.

1) *Bedforms significantly affect hydraulic roughness, much more than skin drag. However, the numerical model employed in this study accounts only for grain roughness (i.e., skin drag) and does not include form drag. As bedforms develop, the local shear stress distribution changes, which could in turn affect the overall area of influence, i.e., the central result of this study. It is important to note that the authors do not model sediment transport (i.e., Exner equation), which may be a major limitation when considering the TKE discussion and the applicability of their conclusions. In this regard, the discrepancy between the observed extent of bathymetric change (i.e., the train of dunes) and the modeled area of increased TKE (lines 162–166) may point to a fundamental limitation of the model. I wonder whether the formation of the bedforms itself altered the local shear stress enough to halt their further development [1]. If so, the length of bedform development could be physical and not a limitation of the experiment run time. In this case then the results obtained via the TKE method may not be fully reliable, and the main quantitative conclusions would be less robust. Note that the TKE measurements and comparison are limited to 13.6D. It is unfortunate that the authors were unable to monitor the temporal evolution of the bedforms – using a moving sonar system for example. I believe that reaching equilibrium in bed evolution is an important prerequisite for drawing meaningful conclusions.*

Reply: You are correct that only the skin friction component of the bed roughness is included in the model setup – this is because the resolution of the model is high enough to resolve the ripples and *implicitly* create the form drag which you mention. The bedforms effect on the flow is visible in the green subpanel of figure 2. We have tried to clarify this in the manuscript, but include a figure here showing the time-averaged dynamic pressure just above the bed, which shows the effect of the ripples are being resolved by the model (Figure below). For cases where the natural state of the bed are larger dunes, the field data which we have included in the revision of the paper represents this case well.

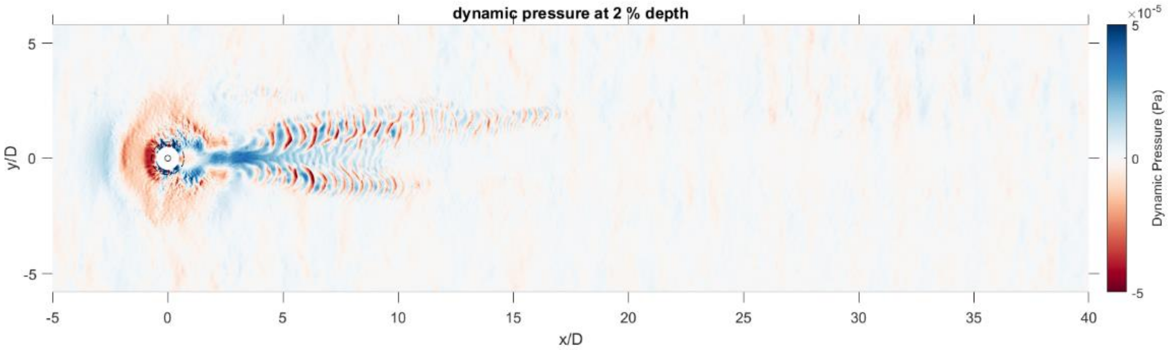

Time-Averaged dynamic pressure near the bed (5th sigma layer, approximately 0.013 cm above the bed).

Our new field data also demonstrate a mature bed evolution, as the wind farm was commissioned in 2009. Our field example has produced quite a long (200 m, or 42 x/D) alteration to the bed - substantially longer than the effect observed in our laboratory experiments. This length is likely to have been caused by both the turbulent wake (the process we are investigating here) under much stronger tidal conditions than we investigated in the lab, but also the particular sediment transport regime at this site – which is sandy bedforms over a gravel substrate. Here we believe (and our ADCP evidence demonstrates) that the enhanced bed shear stress in the flood tide turbulent wake is suspending the sandy sediment, but not the gravel. This is both removing the sand in the wake, but is *probably* also creating a "sediment transport shadow" effect due to reduced supply of sediment further downstream. Whilst our new field example demonstrates the inherent complexity in prediction of sediment transport and bed morphology, we believe that fundamentally it demonstrates that turbulence is the key driver of seabed modification by offshore windfarms. Whilst the example we have used could be considered fairly unique, we have used it because of how well the morphological response in the field illustrates the 3D wake flow structure we have seen in the laboratory and numerical model (Figures 1-2)

2) *From literature (e.g., [2], [3]) —and as also acknowledged in this study— we know that the local scour around a cylinder and the formation of bedforms are initiated by coherent flow structures shed by the obstacle (e.g., horseshoe vortices). These structures are strongly influenced by the Reynolds number (e.g., [4]). According to Table 1, the Reynolds number used in the experiment are approximately an order of magnitude lower than those from the field example used, and it's reasonable to assume higher ranges of Reynolds number in the field. How does this discrepancy affect the results and thus how scalable are the observation of this specific case to the shelf-scale locations used by the authors in their discussion?*

Reply: In many ways this is pointed to an important question - can scaled models represent their large (real world) scaled counterparts? This question is a constant source of discussion, and we rely on the decades of work which have gone into scaling laboratory experiments (e.g., Whitehouse 1998; Frostick et al. 2011).

The field example listed in Table 1 has been updated to a more representative example – I cannot remember why we used such a high flow example here in this table – so it has been updated to the flow state which is more comparable to the mobility of the sediments in the laboratory experiment. This has resulted in a Reynolds number roughly half that previously stated (to 3.62 E+6), which is still an order of magnitude larger than the laboratory experiment. Crucially both laboratory and field example values are high enough that eddy shedding is the dominant frequency in the turbulent spectrum, and above the drag crisis. Whilst there is a difference, the *processes* are as similar as a scaled model could be.

The eddy shedding frequency in the numerical model has been measured at 1.5 m downstream of the monopile (at y = 0, and the free surface) and show a clear peak at 0.166 Hz, which is excellent agreement with prediction of the Strouhal number; which for a mean flow of 0.24 m/s, and a pile diameter of 0.25 m, is 0.1729. Our field example, using the updated Reynolds number should have a Strouhal number between 0.19 and 0.2.

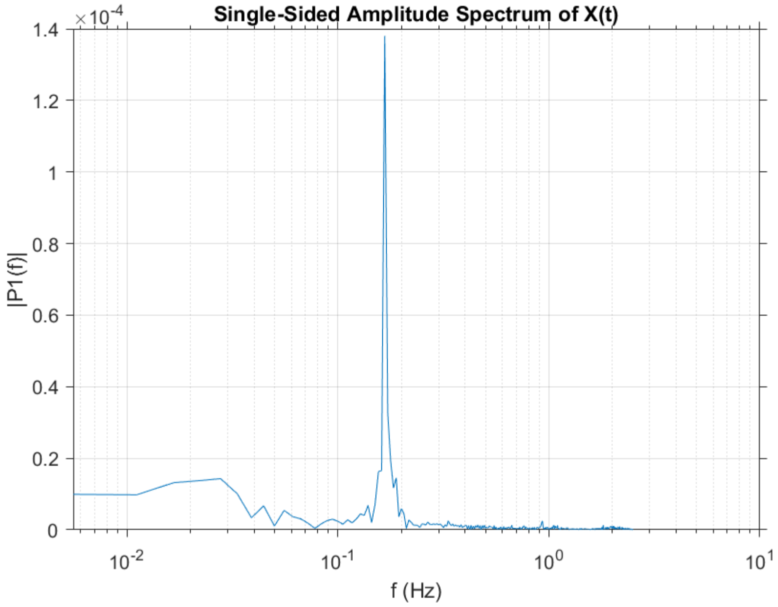

Fourier Transform of the u component, taken at the free surface, 1.5 m downstream of the monopile in the numerical model.

3) *The choice of sediment size is justified to achieve similarity in the threshold of motion, which is a fair experimental assumption and premise. However, the ratio D/d (where D is the tower diameter and d is the grain size), also referred to as the relative coarseness in [5], has a direct impact on the scour magnitude. This was shown theoretically for both bridge pier [5] and hydrokinetic turbines [6] scour predictions. If the magnitude of the scour changes according to different D/d, it might as well affect the consequent bedforms generation and the actual extent of the overall influence. This adds to my doubt that the results presented may be case-specific and the scalability may not be straightforward.*

Reply: This is an interesting point. The scour effects from relative coarseness (ratio D/d50) have been summarised in Section 7.2.6 of “Scour at Marine Structures” (Whitehouse 1998) the scour depth will not be limited by the grain size if D>50 x d50.

Ratio of our field example D to d50 = 4700 mm/0.245 mm = 19184

Ratio of laboratory experiment D to d50 = 250 mm/0.275 mm = 909

Whilst the ratios are very different they crucially both exceed 50 to 100 which is the critical value based on empirical laboratory evidence so as not to have a limiting effect on local scour. Given these ratios the local scour will be controlled by the flow intensity and not the relative coarseness. Therefore, a scaling based on threshold of motion is appropriate.

Regarding the consequent effects on bedform generation, given these ratios it will not be controlled by the relative coarseness but purely by the sediment mobility regime. Our view is if the sediment in field and model scale is at about the same relative sediment mobility, then the mobility in the wake should be similarly enhanced. The exact type of bedforms that will be generated may vary (e.g. ripples or dunes) due to sediment grain size and mobility, but also the change in scale. For the field example, we predict ripples superimposed on dunes, based on the grain size and sediment transport regime. Our bathymetry data for the field

example is not high resolution enough to confirm this (0.5 m resolution), but we have seen this superposition of ripples on dunes in camera observations at nearby locations.

4) *In Table 1, the reported value of θ'_{SF}>/θ_{crit}> exceeds 1 for the Field Example, indicating that sediment substrate is mobile and the conditions are not subcritical in the field. Unless I'm misinterpreting the table, this seems to contradict both the experimental setup and the authors' claim in the introduction that field conditions are subcritical. While this inconsistency may not be a critical flaw since clear-water scour studies remain relevant, it does further question the representativeness of the results at the field scale, particularly given that the proposed numerical model assumes a fixed bed and does not simulate sediment transport.*

Reply: Good point, I cannot remember why we used a field example of 0.60 m/s. We have replaced the field example in table 1 with similar conditions measured in the field, 0.34 m/s flow speed. For this condition the sediment mobility is much more comparable to the laboratory experiment:

$\theta'_{SF}/\theta_{crit}$= 0.67 (field) vs 0.58 (lab)

These are critical points that the authors should address before this work can be considered for publication. I encourage the authors to perhaps incorporate additional analysis, where possible, or discussions to better support the scalability of their findings and strengthen the broader implications of the study. In parallel, clarifying the limitations of the modeling approach and resolving inconsistencies would enhance the manuscript's impact. I look forward to the authors' responses.

Reply: We hope you will find our inclusion of recent field surveys, and the comparison of using a 2D depth mean vs TKE method of obtaining a bed shear stress a compelling example of the significant of turbulence in the wake of the monopile, and demonstration of the method at scale across a range of flow conditions (in this case over a large spring-neap cycle).

Additionally, I hope the following minor comments could help improve the quality and significance of the paper:
- There is a substantial body of research on the large-scale effects of marine energy technologies—such as hydrokinetic turbines and wave energy converters—on erodible bathymetry, sediment transport, and associated ecological impacts. Given the similarities in deployment environments and the nature of the direct impacts, it may be worthwhile to briefly review and reference this literature. This is a minor suggestion and ultimately a matter of the authors' discretion.

Reply: We certainly agree there are similarities to hydrokinetic turbines but wish to keep the manuscript focused on the results we have presented.

- Line 28: missing "r" in the word "Through".

Reply: thank you

- Lines 133 – 135: how was this (i.e., the length of mobile bed underestimation) assessed? is it shown anywhere?

The outline of the bedforms generated in the lab is shown in Figure 1a, and a comparison can be made with Figure 1d, using the 270 micron contour

- Line 137: unnecessary comma at the end of the line?

Reply: thank you

- Line 168 – 170: I think the development and evolution of the turbulent structures responsible for the local mobility of send in the presence of obstacles should be described better and upfront for the general reader to understand. This might imply restructuring the first two main subsections of the Results section and explain the turbulent processes before showing bathymetric and TKE results. Again, this might be ultimately a stylistic decision.

Reply: This is indeed a reasonable suggestion, and one we spent time considering. In our paper we highlight the importance of turbulence on sediment transport, yet we only show that turbulence in a time-averaged manor and are not showing the beautiful detail of turbulence that many papers do. We have decided to keep this approach, due to two reasons. 1) the paper is long enough, whilst being quite focused in its remint, and 2) we are using the spatial distribution and intensity of TKE to describe and explain bed mobility in both the laboratory, laboratory model and now in the field example we have added to the manuscript – to be consistent across all three we need to keep to a time-average presentation of turbulence. We will try to include figures and video of the turbulence in presentations!

- Line 229 – 230: "bed shear stresses high enough to mobilize sediment under these "below threshold" conditions thus forming the bedforms". I think this might be misleading because the conditions locally go indeed over the threshold. Maybe it can simply be reworded as: "The resultant three-dimensional wake flow generates near-bed turbulence that locally increase the shear stress over the threshold of motion thus forming the bedforms."

Reply: This is a good suggestion, which we have used in our revision.

- Figure 1c: I was surprised to see such a pronounced upwelling region in front of the monopile, as previous studies have shown that flow typically moves downward when encountering a cylinder, generating a recirculation zone and horseshoe vortices around the base. I assume this upwelling is the result of the specific upward-sloped design of the rock armor at the pier's base—can you confirm if this is the case? I believe it would be worthwhile to expand the description of Figure 1c when discussing the flow development and turbulent structures around the monopile.

Reply: Yes, this is indeed due to the rock armour, we have expanded this description of Figure 1c. Including realistic rock armour was important for the design of the experiment to try and make it as true to real world examples as possible for a scaled laboratory experiment:

"Upstream of the monopile, the slope of the rock armor produces substantial upwelling before the flow reaches the monopile, demonstrating that the specific design of rock armor scour protection will influence near monopile flow structure.".

- Line 462: missing "/" between x and D.

Reply: Thank you.

- Line 504: something is missing at the end of the sentence.

Reply: Thank you.

- Line 520: I assume it should be “altitude” not “attitude”. Also, why was the accurate control of the instrument’s elevation not possible?

Reply: We have changed “attitude” to orientation relative to the horizontal, as “attitude” is more related to an orientation relative surface of the planet. We couldn’t guarantee accuracy of the orientation of the instrument to within 1%, so therefore as the error when calculating Reynolds stresses increases substantially from even an error in orientation of 1%, we have forgone this method.

- Streamwise component of momentum equation (second question of Eq. 10): should be u and not i before ∂u/∂x.

Reply: Thank you. Equation typos are my least favourite.

Reviewer #2 (Remarks to the Author):

This paper presents the results of laboratory experiments and numerical simulations of the flow and bed disturbance around a protected wind turbine monopile. The experiments are original in that they provide novel data on the flow far downstream in the wake of the pile, alongside the presence of scour protection at the base of the pile. The numerical simulations are state of the art, based on the 3D version of OPENTELEMAC. The experimental data were used to validate the numerical simulations in the wake, in terms of both mean velocity and turbulent kinetic energy (TKE). The numerical results are then used to extrapolate the region of influence of the monopile in terms of bed mobility. The authors address three research questions: (1) What is the intensity and spatial scale of the hydrodynamic effects on the bed from a scour-protected monopile, accounting specifically for turbulence and not just mean flow? (2) What is the change in sediment transport capacity in the wider area away from the monopile when the seabed sediment is naturally immobile but close to the mobilisation threshold? (3) How might this consequently affect habitat composition and distribution, and its prediction? While the first question is addressed in the article, the second relies heavily on the model, for which additional details would be needed, and the third is very speculative, as no data are used to infer habitat modifications. I would have expected to see some numbers here! In my opinion, the technical part of the manuscript is interesting and could be published, provided additional information on and verification of the numerical simulations are provided. The third question is not mature enough at this stage and would require much more work, which I don't believe could be achieved through a major revision. Therefore, my recommendation would be to reject the manuscript, but encourage the authors to resubmit, potentially to a different journal.

Reply: We are glad you find the technical aspects of the work interesting and hope you will find the added details to the modelling satisfactory. After reflecting on the editors and reviewers’ comments we have included recent field data into the revised manuscript which demonstrates the turbulent wake effect of increasing bed shear stress at an operational offshore wind farm. We believe this new field evidence adds validity to our new method for real world conditions.

Whilst we cannot assess the exact impact that these turbulent wakes are having for every offshore windfarm in the world, we can at least present the potential scale of the impact – which is what we have tried to do with our shelf-scale estimation. We do not attempt to say that these scales are exactly what will happen – but that this is a process which needs to be taken into account when designing this renewable infrastructure. For the impacts to marine life – we have removed this as one of our aims, as any habitats modelling and quantification of the effect in the laboratory experiment would be hypothetical, and at our field site specific – as such we have changed aim three to "how might accelerated seabed mobility affect seabed composition when the bed is already mobile?" which is our new field case (Figure 3a). We have attempted to reinforce the linkages between physical environment and habitats. Variables like temperate, depth and, of importance in this paper, sediment grain size and bed shear stress are key variables in the EUNIS classification scheme. As you say, adding in a section of work showing these changes for our field site is essentially another paper entirely.

We hope you find our revised, and focused revision acceptable.

Comments:

1) The predicted bed shear stress from this blended method shows the wake of the monopile to be dominated by TKE —> show the equation

Reply: This has been included as Equation 11 in the methods section

2) Fig 1: You normalize y and x but not the elevation. Why ? Zoom in on the region around the pile to illustrate the flow around the structure (panel b : bed shear stress) how do you measure the bed shear stress ?

Reply: The z elevation was not normalised as a stylistic choice. The point of the figure is not to show the flow structure at the monopile in a lot of detail – this has been published many times before - but to focus on the turbulent wake. The method of measuring bed shear stress in the laboratory, and from the numerical model is described in the method section, but briefly, we used a Nortek Vectrino profiler in the laboratory to measure the near bed mean flow and turbulence, and we calculated the bed shear stress via the method of Soulsby, wherein the TKE at 10% of the flow depth was multiplied by a constant (0.19) to produce a bed shear stress.

3) Which turbulence model is used in the simulations and how are the boundary conditions set, especially on the cylinder? What is the mesh resolution in the x, y and z directions around the cylinder and near the bed? I found the answers in the supplementary material, but it is disconcerting to see the results without knowing the underlying modelling assumptions. TELEMAC3D is not known to work very well for simulating flow around cylinders, at least in the near field. It is important to provide many more comparisons of the flow in the near and far fields in order to support the use of the model for extrapolating measurements.

Reply: We do understand your frustration, the paper is ordered this way to conform to the Nature publication format standard. TELEMAC-3D has been shown to work well around cylinders using the turbulence model we have used (Spalart and Allmaras 1992), with high enough resolution at the monopile. Following (Bourgoin 2019; Bourgoin et al. 2021; 2020), we used a monopile resolution of 2.4 mm. The lowest sigma layer is 2 mm above the bed. We hope the comparison of laboratory measured and numerical modelled bed shear

stresses shown in Figure 8 is convincing. There is also a comparison of the profiles of U,V,W and TKE in the supplementary section.

4) l. 349-356: This is highly qualitative and speculative. Can you quantify these arguments, for example by giving typical values of the turbidity levels in the wake and comparing them with those outside the wind farm?

Reply: The purpose of this paragraph is to discuss the implications of offshore wind farms on seabird foraging strategies. The papers cited all have different measurements of the environmental conditions (e.g. Secchi Depth); there is not one universal method as this is a recent and evolving subject. We have changed to language in this paragraph to make sure we are not saying that our manuscript is showing changes in suspended sediment concentration. Our work sits alongside others in trying to highlight the range of effects on seabird foraging strategies that offshore windfarms can have - i.e. it is not just purely a negative effect for all seabirds and we believe the environmental impact assessments of offshore windfarms needs to take a more holistic view.

5) An important issue with this work is that all these quantities are obtained from parameterisation in the numerical simulation, and there is no experimental data to support the estimation of bed shear stress based on hydrodynamics, except for bed elevation.

Reply: We believe the reviewer is incorrect with this statement. We report extensive large-scale laboratory measurements of bed shear stress that have been obtained using robust and rigorous methodologies and these provide the foundation upon which the numerical model is developed; they are compared with the numerical model in Figure 8. Unless a numerical model is a direct numerical simulation of all of the scales of turbulence, then it will have parameterisation – this is the vast majority of numerical models used in environmental sciences and they are well supported by validation against observation.

6) Table 1: Table 1: Why use the same particles in a scaled model? Sediment dynamics will differ significantly; should the author not use a Shields similarity to match bed mobility?

Reply: Good point, I cannot remember why we used a field example at 60 cm/s. This table has been updated with representative flow conditions.

7) The measurements do not cover the 25D extension of the wake therefore the conclusion of the article is an extrapolation based on the simulation. This result cannot be fully trusted until the simulation has been validated more carefully.

Reply: We believe the validation of the model is excellent as demonstrated by figure 8 and the model validation supplementary section. If we cannot use model data outside of the validated locations, then not a single meteorological or oceanographic model would be valid. Given the validation shown in Figure 8 in the manuscript and in the supplementary material, we are confident that the model is representing the *processes* very well.

8) How can the wake be accurately represented if the no-slip boundary condition on the monopile is not accounted for? How are the BCs for turbulence specified? How is vortex shedding predicted? The authors need to present results on wake dynamics from the

simulations, such as the Strouhal number, as well as some snapshots of coherent vortices shed from the cylinder.

Reply: The monopile uses a no-slip condition at the monopile, with zero roughness. This has now been clarified in the methods section. The turbulent wake in the model had a dominant eddy shedding frequency of 0.166 hz, giving a Strouhal number of 0.172, which compares very well with the prediction

9) A domain covering a sandy section of the flume experiments (3 m x 16.2 m) was created with a resolution of 50 mm (see Figure 4d). This is relatively large compared to the thickness of the boundary layer (BL) around the monopile.

Reply: As per the methods section, the resolution of the horizontal mesh at the boundary of the model is 50 mm, but there are multiple regions of higher resolution within this to properly resolve the turbulent wake, see figure below:

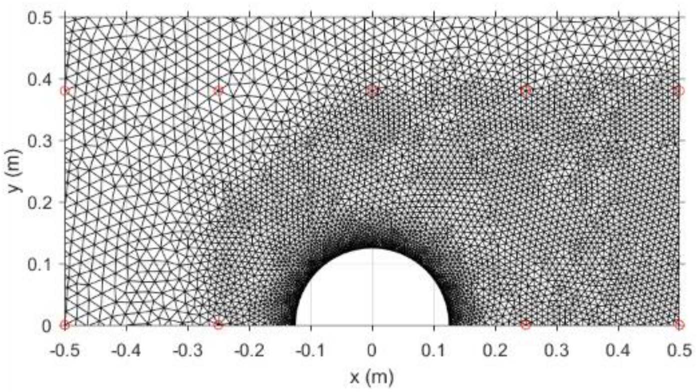

Example of the unstructured triangular mesh around the monopile. Red circles denote laboratory measurement locations which are fixed points in the numerical mesh. Flow direction is left to right.

Vertical layering the model is described in the supplementary material, but we have included the key figure here:

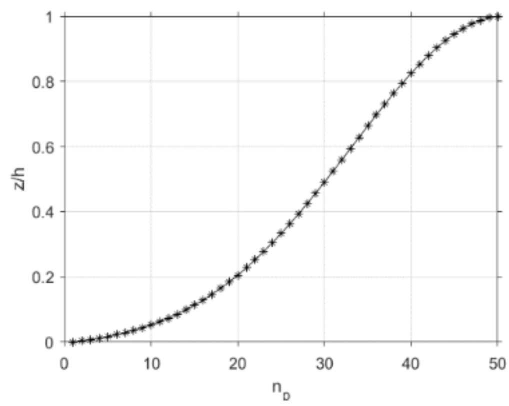

Vertical spacing of the sigma layers in the model, with normalised vertical coordinates (z elevation / depth) and sigma layer numbering from zero (the bed) to the surface (50)

10) « Flow depth and discharge were specified at the inlet, with a power law profile specified

in the vertical. The flow depth and discharge at the inlet was fixed at 0.6 m and 0.435 m3 s-1, respectively. The outlet was given a fixed discharge of -0.435 m3 s-1, and a free depth to account for the water surface slope »: The way the boundary conditions are set here doesn't make sense if the flow is subcritical. Please explain.

Reply: For each iteration the free surface is first solved using the wave equation, then the velocities are solved. The velocity at the boundary will then be scaled to give the prescribed outflow discharge whilst maintaining the cross-sectional shape of the flow field.

Typo: l.571: missing 0: 1^... --> 10^...

Reply: Thank you, this has been corrected.

Reviewer #3 (Remarks to the Author):

Unsworth et al. present a fascinating manuscript on how offshore wind turbines can substantially alter the seabed. As my expertise is in marine ecology (and not physical oceanography), I focus my review on the ecological aspects and implications from the manuscript.

Overall: The authors' finding that the seabed sediment is altered up to 25x the monopile foundation diameter downstream is a novel finding that has the potential to change how we think about potential environmental. impacts of offshore wind energy. Additionally, the manuscript is particularly timely as, especially in the U.S., there has been a high degree of uncertainty regarding the level of impacts from offshore wind energy development. Unsworth et al. helps resolve some of this scientific uncertainty. I suggest that the manuscript be considered for acceptance to Nature Communications following a minor revision.

I've included several comments that the authors wish to consider addressing:

While I realize that the study focused on a single turbine, I suggest that the authors consider a bit more about what may happen with multiple turbines in the same wind farm. The calculations in Appendix 1 that were also verbally stated in the results/discussion of what to expect with a given number of turbines were really helpful. However, in the U.S., monopile foundation turbines are typically spaced about 1 nmi apart (I'm not sure how this differs in Europe and Asia), but I am keen to understand how the turbulence fields associated with multiple individual wind turbines may interact with one another. In particular, would the authors suggest that the magnitude of impacts may increase through interactions of turbulence and mean flow from nearby wind turbines, including those upstream, downstream, or positioned laterally? This would likely be a hypothetical statement, but I think mentioning the potential for interactive synergies could be worthwhile, especially from an ecological perspective. This is because wind turbines present such a unique configuration of artificial structure in the ocean given that hundreds of turbines are often installed in proximity to one another within a given wind farm.

Reply: it is certainly possible for the turbulent wakes emanating from offshore wind farm monopiles to interact. This interaction can be “local” for individual wakes and this may well

lead to amplification of turbulence at the seabed. There are also possible larger scale "group" effects where the turbulent wakes act as a roughness to the flow – channelling and accelerating the tide into channels between monopiles. A recent paper by Carpenter and Guha (2024) presents some good examples of this process.

For your example of a windfarm with 1 nmi distances between monopiles the turbulent wakes will scale with the monopile size, and flow conditions. For similar flow conditions to our laboratory experiment we would predict an amplification of bed shear stress up to 24 monopile diameters downstream.

This may also be hypothetical, but I'm curious – do the authors think that similar processes may occur around floating turbines? It could be interesting to mention this as a future research direction, especially in deeper waters where floating turbines are being considered (or are already being installed!).

Reply: The question of floating turbines is an interesting one. We have colleagues working on this on a different project at the moment. The main impact from floating turbines *appears* to be on stratification – which will be very important for marine life and potentially the carbon cycle (ventilation) in shelf seas. As this is certainly beyond the remint of this paper and as such we have not discussed it in text, but we do believe that alteration of the seabed due to offshore wind farms could happen due to the action of the anchors and chains on the seabed - similar effects have been seen near harbours (Watson et al. 2022). Depending on substrate and marine life, these scours could be important.

Marine spatial planning - Marine spatial planning is mentioned in the abstract and in a few other locations. If space permits, I would encourage the authors to describe their vision more fully for how their findings should be (or could be) incorporated into marine spatial planning frameworks. The study findings could be quite catalytic for marine spatial planning, and for the calculation/estimation of cumulative impacts.

Reply: We welcome the opportunity, and have added the following to the end the discussion in our manuscript:

"Better forecasts of seabed sediment mobilization and the extent to which it can alter the distribution of benthic habitats can strengthen marine spatial planning. The work presented here will help in the development of robust, multi-scale quantifications of seabed sediment dynamics in places where new morphodynamic feedbacks are introduced by seabed infrastructure which we show can amplify bed shear stress and resuspension well beyond the immediate scour zone. We advocate for our method of integrating field observations and process-based models as a robust way to anticipate environmental thresholds with confidence and guide evidence-based marine spatial planning to safeguard carbon-rich seabed sediments and their habitats, and the infrastructure we increasingly rely on."

Scour protection design – I see potential application of the study findings to help industry/developers better design scour protection. Are there general recommendations that you would have for developers on how to best design scour protection to minimize turbulence (if that is the goal…)?

Reply: The general recommendations for the design of scour protection are to use the smallest rock grade that provides the stability required, to minimise the roughness generated

turbulence, and to minimise the thickness and lateral extent of the scour protection to minimise blockage of flow and sediment transport. Both aspects can be evaluated in desk study-based designs supported by numerical modelling (Computational Fluid Dynamics) and physical modelling of the type carried out in our paper.

Line 60 – Consider clarifying the 80%. Is that 80% of turbines globally that are monopolies? And, did the citation for the 80% value appear earlier in the sentence?

Reply: we have moved the references to the end of the sentence for clarification, thank you.

Line 66-68 – Suggest clarifying this sentence. I had trouble following it.

Reply: we have attempted to clarify this sentence: "Existing quantifications of the recovery distance of the mean flow downstream of a monopile tend to focus on the flow directly behind the monopile (at a distance between 2 and 15 monopile diameters, *D*)[27,28], but not in the broader area behind the monopile."

Line 94-95 – Is there a particular threshold for "deep" here? I ask this because floating turbines are being considered in deep waters instead of monopiles, in some cases (and many cases in the U.S.).

Reply: Good question, several years ago 30 m was considered deep for monopiles, now we are seeing proposals for monopiles in 50-60 m of water, sometimes with a conical shape (larger at the base). We omit floating offshore here, but we do think there are potential seabed affects from floating windfarms due to the anchors and cables – that is for another project!

We have added (30 m +) to clarify this in the manuscript.

Line 361-362 – The sentence on artificial reefs is a bit misleading. I suggest clarifying. Artificial reefs are typically considered to have a mixture of unintended ecological consequences and – in some cases – ecological benefits.

Reply: Good suggestion, we have replaced this sentence with the following:

"Removing such infrastructure, and thus returning the seas to their prior "natural" state (which is often required by law, e.g. following OSPAR Decision 98/3), might not always be the best option for marine ecosystems (Knights et al. 2024; Clare et al. 2025). So far, the primary focus of the discussion has been on the role of infrastructure as artificial reefs (Lemasson et al. 2024) and the resulting mix of what some perceive as desirable (e.g. enhanced diversity) and undesirable (e.g. a vector for invasive species) ecological consequences. Here, we show that alteration to seabed mobility, and potential for seabed modification which can change the distribution of benthic habitats, are additional potential effects associated with the presence (or removal) of infrastructure, and help underpin the need for more nature positive approaches (Bull et al. 2019; Maron et al. 2023). "

Thank you for the opportunity to review your manuscript!

Reply: Thank you so much for taking the time to review it!

References:

Bull, Joseph W., E. J. Milner-Gulland, Prue F. E. Addison, et al. 2019. ‘Net Positive Outcomes for Nature’. *Nature Ecology & Evolution* 4 (1): 4–7. https://doi.org/10.1038/s41559-019-1022-z.

Carpenter, Jeffrey R., and Anirban Guha. 2024. ‘Blocking Effects on Mean Ocean Currents by Offshore Wind Farm Foundations’. *Physical Review Fluids* 9 (10): 103802. https://doi.org/10.1103/PhysRevFluids.9.103802.

Clare, M.A., A.R. Gates, D.O.B. Jones, et al. 2025. ‘Environmental Considerations for the Decommissioning of Subsea Cables’. *Journal of Environmental Management* 396 (December): 127962. https://doi.org/10.1016/j.jenvman.2025.127962.

Frostick, Lynne E., Stuart J. McLelland, Theresa G. Mercer, and J. Kirkegaard, eds. 2011. *Users Guide to Physical Modelling and Experimentation: Experience of the HYDRALAB Network*. Online-Ausg. IAHR Design Manual. CRC Press/Balkema Book.

Knights, Antony M., Anaëlle J. Lemasson, Louise B. Firth, et al. 2024. ‘To What Extent Can Decommissioning Options for Marine Artificial Structures Move Us toward Environmental Targets?’ *Journal of Environmental Management* 350 (January): 119644. https://doi.org/10.1016/j.jenvman.2023.119644.

Lemasson, Anaëlle J., Paul J. Somerfield, Michaela Schratzberger, et al. 2024. ‘A Global Meta-Analysis of Ecological Effects from Offshore Marine Artificial Structures’. *Nature Sustainability* 7 (4): 485–95. https://doi.org/10.1038/s41893-024-01311-z.

Maron, Martine, Fabien Quétier, Mariana Sarmiento, et al. 2023. ‘“Nature Positive” Must Incorporate, Not Undermine, the Mitigation Hierarchy’. *Nature Ecology & Evolution* 8 (1): 14–17. https://doi.org/10.1038/s41559-023-02199-2.

Nezu, Iehisa, and Hiroji Nakagawa. 1993. *Turbulence in Open Channel Flows.* IAHR Monograph. Balkema.

Watson, Sally J., Marta Ribó, Sarah Seabrook, Lorna J. Strachan, Rachel Hale, and Geoffroy Lamarche. 2022. ‘The Footprint of Ship Anchoring on the Seafloor’. *Scientific Reports* 12 (1): 7500. https://doi.org/10.1038/s41598-022-11627-5.

Whitehouse, R.J.S. 1998. *Scour at Marine Structures: A Manual for Practical Applications.* Thomas Telford.

REVIEWER COMMENTS

Reviewer #1 (our responses in green):

Dear authors,

I have reviewed the revised manuscript together with the authors' responses to my comments on the original submission. The authors have responded satisfactorily to most of my comments, and I believe this has resulted in a substantially improved manuscript. I particularly appreciate the addition of the new field data, which further reinforces the authors' central argument that wake turbulence (measured while accounting for wake-added TKE) is the main driver of bathymetric impacts that develop over much longer distances than typically considered.

As a result, my original concerns regarding the specificity of the results have been partially addressed, and I believe the revised manuscript now presents much stronger and more compelling arguments. I still believe—and note that this concern was also shared by Reviewer #2—that many of the arguments and conclusions related to potential ecological implications at the shelf scale remain somewhat speculative. However, I also consider this acceptable, as these discussions naturally stem from the core results of the work (i.e., the extended bathymetric effects observed both experimentally and numerically). These are framed as potential consequences, and I am therefore comfortable with this level of speculation.

That said, a few comments remain:

1) I do not fully understand the reply to my comment #1. The authors state that "the resolution of the model is high enough to resolve the ripples and implicitly create the form drag which you mention." Are the authors using the laser scan from the experiment as a DEM for the numerical simulations? If so, I may have missed this detail in the methodology, and I suggest clarifying it.

- Reviewers 1&2 seem to have missed this detail, so we have altered the text to clarify this for the reader: Starting line 117; "During the experiment we measured the mean flow and turbulence downstream from the monopile and collected 3D topographic data of the bed before and after the experiment with a laser scanner at a resolution of 1 mm. Using this information we then created and validated a three-dimensional (3D) computational fluid dynamics (CFD) model using TELEMAC-3D, which includes rock armor and bedforms in the model bathymetry."

2) I disagree with the authors' response to my comment #3. I reviewed Section 7.2.6 of Scour at Marine Structures (Whitehouse, 1998), which is cited by the authors, and I understand the premise of their argument. However, this interpretation is in direct disagreement with the data presented in Figure 4 of (Manes and Brocchini 2015, please see figure in the attachment), which clearly shows that dimensionless scour depth varies for values of the ratio D/d□□ well beyond 50.
The data combines several (more recent) studies: (Ettema 1980; Sheppard et al. 2004; Ettema et al. 2006; Lança et al. 2013).

While I agree that scour is primarily controlled by flow intensity and that scaling based on the threshold of motion is appropriate, I also believe that other parameters—such as relative

coarseness—play an important role. This is physically intuitive, and recent literature supports this view. Even if the effects on bedform generation might be minor, I believe the statement in the authors' response that "the local scour will [not] be controlled by […] relative coarseness" is conceptually incorrect.

I therefore encourage the authors to further review the relevant literature (perhaps starting with Manes & Brocchini, 2015) and to expand or refine their discussion accordingly. These issues directly relate to my original concerns regarding the scalability of the results. While I appreciate the revised wording used by the authors when applying their findings to the field scale (e.g., "Using our laboratory results as a guide rather than the field evidence…"), I still believe the authors should explicitly discuss scalability in terms of the main physical parameters (e.g., grain size distribution, relative coarseness, Reynolds number, etc.). In this regard: what uncertainty is associated with the predicted affected shelf areas (km^2) of their real world examples? I suggest that these aspects be discussed either in the methodology section or, at minimum, in the Supplementary Information.

We do agree with the reviewer's point that our shelf scale numbers are estimated in the parameter space of our laboratory experiment. We believe the estimate is relevant in its order of magnitude. This is the first quantification of its kind, and is the baseline that will be refined by future work across environmental conditions. We have now added the following to the end of the paragraph which provides better context to our estimates (Line 357):

"The area of amplified bed shear stress produced in our laboratory experiment represents one hydro-sedimentary condition across the spectrum of tidal and sedimentary regimes. The exact effects on any windfarm in the world will depend on local hydrodynamic and sedimentary conditions, which require modelling validated with local observations. Factors such as Reynolds number and roughness of the natural seabed and/or scour protection are likely to scale the area affected as there are phenomenological similarities with monopile scour(Manes and Brocchini, 2015)."

3) I understand the authors' intention to keep the manuscript focused on wind energy installations, and I acknowledge that this is a reasonable choice. However, statements in the article such as "While scour adjacent to infrastructure has been studied extensively, the impact on sedimented seafloors further downstream has been overlooked, despite its importance for environmental risk assessment" are not accurate. Given that the scour process and its associated downstream impacts on sedimented seafloors are phenomenologically similar, it is misleading to claim that such downstream impacts have been overlooked (at least by the scientific community). This statement risks disregarding a substantial body of prior work (some examples: (Neill et al. 2009; 2012; Fairley et al. 2015) and more for tidal environments, or (Musa et al. 2018) for river environments). I suggest either acknowledging this existing literature or revising the statement accordingly.

We agree with the reviewer. In the work cited above, the changes to sediment transport are due to alterations of mean flow speeds, rather than the 3D turbulent flow structure which we detail in our work. We have therefore changed our statement to emphasise our focus on the effect of the turbulent wake and sediment transport capacity:

"While scour adjacent to infrastructure has been extensively studied, the potential for changes to sediment transport capacity further downstream due to turbulent wakes has remained largely unexplored despite seabed composition being a critical driver for habitat suitability.."

4) Lines 317-326: It may be difficult for readers to understand the distinction between "non-

wake” and “wake” conditions (which depend on tidal direction) based on the current text and Figure 4 alone. I suggest briefly explaining this distinction here to improve the transition at line 317.
- we have now clarified this in the manuscript in lines 317 to 326

Additionally, the general readership of Nature Communications may not be familiar with bedform regimes and transitions (Figure 4 and the associated discussion). It may therefore be beneficial to provide additional explanation, unless this is already addressed elsewhere in the manuscript (in which case I apologize for overlooking it).
We considered this to be a great idea, and we have added an illustrative cartoon on the different sediment transport stages to panel f of Figure 4 to make this clear to the reader

In conclusion, I would like to emphasize that none of the points raised above constitute major flaws, and I continue to believe that the study has merit and is suitable for publication in Nature Communications.
- thank you once again for your time and your constructive reviews.

Reviewer #2 (our responses in green):

Comments:

1) I won’t insist on the depth normalization. I do believe that the bead shear stress data upstream the pile would be interesting and they could have been added to the supplementary file « Model Validation ».

This is in the paper: see Line 151

“The background bed stresses measured upstream of the monopile (indicated in Figure 7d of the paper) were measured to be 0.1257 N m^{-2}”

2a) I understand that the format of the Nature paper does not allow the details of the model to be introduced in the main text. However, to make the work fully reproducible, it is important to clarify how the boundary conditions for nuTilda are specified for the smooth cylinder wall and rough bed.

TELEMAC is a little different to other CFD codes in that there’s no explicit way of setting a boundary condition for nuTilda in the model setup files; the user sets the roughness regime of the walls as either “rough” or “smooth”. Roughness values in TELEMAC 3D are given in the sand grain equivalent Nikuradse roughness, and can be specified in the model set up file (called the steering file “.cas”), or a user can specify a spatially and/or temporally varying roughness if the model requires it. This information is contained within the model geometry file (“.slf”), so every wall node can have a unique roughness value. Wall roughness affects turbulence in the model via the velocity profile shape - the calculation of the shear velocity (u*) at a wall uses either the law of the wall (if the regime is set to “rough”) or using a Reichard law of velocity profile when it is set as “smooth”.

This difference in boundary conditions settings (compared to other CFD codes like OpenFOAM) is due to TELEMAC’s focus on modelling environmental flows. Surface

roughness is a crucial parameter in open channel flows and so the code is focused so the user gets those values correct.

The following has been added to the methods section of the manuscript to better explain how turbulence is calculated in the model, and how the boundaries are dealt with:

The one-equation Spalart-Allmaras turbulence model[116] was used as it has been demonstrated to be an efficient and accurate model for flows with adverse pressure gradients, particularly flows around monopiles and bedforms[18,117,118]. In this model the eddy viscosity, ν_t, isn't solved directly Instead, a transport equation for a working variable $\tilde{\nu}$ is solved. From this, the eddy viscosity is computed:

$$\nu_t = \tilde{\nu} f_{v1}, \qquad f_{v1} = \frac{\chi^3}{\chi^3 + C_{v1}^3}, \quad \chi = \frac{\tilde{\nu}}{\nu}, \tag{15}$$

where f_{v1} is a damping function that limits the turbulent viscosity near solid walls, and C_{v1} = 7.1.

"Smooth" boundaries in the model, such as the monopile and the sidewalls, have Dirichlet boundary conditions so that $\tilde{\nu}_{wall} = 0$. Turbulence properties in the nodes adjacent are calculated via the turbulence model based on the velocity gradient, which is calculated using a Reichard law; assuming the first node away from the boundary is in the log-layer.

For hydrodynamically "rough" boundaries in the model (the sand and gravel on the bed), roughness is introduced using the equivalent (Nikuradse), sand grain roughness[119]. The roughness values were calculated using the van Rijn estimate of grain roughness for the sediments (Van Rijn, 2007; $3D_{90}$) used in the laboratory (sand roughness length k_s = 0.00087 m, gravel k_s = 0.036 m). Velocities at rough boundaries are calculated using the law of the wall, using the roughness lengths specified on the bottom boundary. The model therefore assumes the first node above the boundary is within the log-layer. For the Spalart-Allmaras turbulence model[116], TELEMAC 3D uses an effective wall distance:

$$d_{eff} = d + C_{t4} k_s \tag{16}$$

Where d is the distance to the wall, k_s is the sand grain equivalent Nikuradse roughness, C_{t4} is 0.5. The distance d_{eff} replaces d in the destruction term in the turbulence model to mimic the loss of the viscous sub layer in rough wall flows. Several other constants are used in this turbulence model, all are the same as the original publication except: $\sigma = 0.85$, $C_{b1} = 0.105$, $C_{w1} = 2.07$, which have been found to perform better in TELEMAC 3D [121].

Flow was discretized using an unstructured triangular mesh, created in *BlueKenue*[122]. A domain covering a sandy section of the flume experiments (3 m x 16.2 m) was created with a resolution of 50 mm (Figure 7d). Various monopile grid resolutions were tested, and the best results were obtained with the highest resolution, 2.45 mm (see supplementary table S1). Refinement regions upstream and downstream of the monopile increased the mesh resolution from 10 mm to 30 mm in the wake, over the bedforms and rock armor. The 10 mm near-field region was found to be essential to reduce numerical diffusion in the monopiles' turbulent wake.

Wall-distances ($y^+ = yu^*/\nu$), values for the monopile on average are 47, to a maximum of 140. Low values (minimum of 3.1) around found in the flow separation bubbles. Whilst y^+values this low reach into the viscous sub-layer, the low velocities magnitudes (<0.05 m s^{-1}) in these regions should result in little error due to overprediction of shear stress on the wall. Where velocities at the monopile surface are high, y^+ values are between 40-140.

2b) Also, just to clarify, you do not account for the bedforms in the simulation? You use a constant bed roughness everywhere? Did you use the bed scan to build the bathymetry in the model? The bed roughness might play a role in the bed shear stress estimate so it seems important to clarify this point.

- Both reviewers seem to have missed that the laser scan of the bed, and the numerical model's bathymetry, includes the bedforms. We have now clarified this at multiple places in the manuscript as it is an important detail which appears to be easily missed.

3) An important issue with this work is that all these quantities are obtained from parameterisation in the numerical simulation, and there is no experimental data to support the estimation of bed shear stress based on hydrodynamics, except for bed elevation.
--
Reply: We believe the reviewer is incorrect with this statement. We report extensive largescale laboratory measurements of bed shear stress that have been obtained using robust and rigorous methodologies and these provide the foundation upon which the numerical model is developed; they are compared with the numerical model in Figure 8. Unless a numerical model is a direct numerical simulation of all of the scales of turbulence, then it will have parameterisation – this is the vast majority of numerical models used in environmental sciences and they are well supported by validation against observation
---
Let me phrase this differently. You have not explained precisely how you processed the data to obtain the bed shear stress using the mean flow method.

- This is because we do not show laboratory experiment bed shear stresses derived from the mean flow method. Mean flow conditions are specified in Table 1, with equations 1-2 detailing how we calculated the mobility of the sediment.

How do you estimate bed roughness? How much is present at each point? What precise expressions do you use?

- Bed roughness in the numerical model is defined via the Van Rijn method (lines 678-650),

  "For hydrodynamically "rough" boundaries in the model (the sand and gravel on the bed), roughness is introduced using the equivalent (Nikuradse), sand grain roughness[119]. The roughness values were calculated using the van Rijn estimate of grain roughness for the sediments (Van Rijn, 2007; $3D_{90}$) used in the laboratory (sand roughness length k_s = 0.00087 m, gravel k_s = 0.036 m)."

- for mean flow conditions in the laboratory experiment, we use the Van den Berg and Van Gelder, (1993) formulation of the shields number which are shown in equations 1-2 in the paper. Roughness is defined by the Chezy parameter.

You also claim that other methods, such as the Reynolds stress method, are inaccurate by ±10%. The TKE method is also quite indirect. It is important to provide more details on this crucial point to make the analysis fully reproducible. You could provide a comparison in the supplementary material for example. It would also be important, to be fair, to introduce error bars on the laboratory-measured bed shear stress.

  - In the manuscript we cite the following papers which will give a reader a good understanding of errors in obtaining bed shear stresses in laboratory experiments: (Dyer, 1986; Biron *et al.*, 1998, 2004; Kim *et al.*, 2000; Williams, Bell and Thorne, 2003; Pope, Widdows and Brinsley, 2006; Jeon and Kang, 2024). We do not feel it is

necessary to enter a discussion of this point, as it has been well discussed in those papers.

On another note, did you determine the wavelength and amplitude of the bedforms along the two transects shown in the insets of Figure 1(a)? If so, that information would be useful to provide.

- Good point, we add the following to the methods, so this information is available to the reader
  "The bedform detection tool BAMBI (Cisneros et al., 2020) was used to measure bedform geometry from the bed scan, and mean ripple heights ranged between 0.005 m and 0.015 m, with mean lengths a more constant 0.15 m. Lee side angles measured between 25 and 35 degrees.

4) How can the wake be accurately represented if the no-slip boundary condition on the monopile is not accounted for? How are the BCs for turbulence specified? How is vortex shedding predicted? The authors need to present results on wake dynamics from the simulations, such as the Strouhal number, as well as some snapshots of coherent vortices shed from the cylinder.
--
Reply: The monopile uses a no-slip condition at the monopile, with zero roughness. This has now been clarified in the methods section. The turbulent wake in the model had a dominant eddy shedding frequency of 0.166 hz, giving a Strouhal number of 0.172, which compares very well with the prediction
---
In line with comment 4 above, I still think more information is needed on the flow and turbulent boundary conditions (including the effect of roughness), as well as an estimation of the initial grid size at the cylinder in wall units. This will help to confirm that the boundary layers are well resolved. Grid size in SI units is not helpful here. I have seen the FFT plot in the wake and the Strouhal number estimate in response to Reviewer #1. I believe that some of this information should be included in the manuscript. As your work focuses on the wake, including these quantities is essential to strengthening your results. Snapshots of the Q criterion would also be useful, but I don't want to force you to include them.

- We have added the information of eddy shedding frequency to the supplementary material.
- Y_plus units for the monopile have also been added to the manuscript, lines 665-669. The values range from a maximum of 140 and a minimum of 3.1, with a median of 40 and a mean = 47
- With the lower values confined to the flow separation regions with very low U. As the monopile had no roughness in the model, we don't see that resolving the boundary layer down to the viscous sublayer to be an issue, and are overall happy with the averages being >30 and well below the limit of the model (300).

9) A domain covering a sandy section of the flume experiments (3 m x 16.2 m) was created with a resolution of 50 mm (see Figure 4d). This is relatively large compared to the thickness of the boundary layer (BL) around the monopile.
--
Reply: As per the methods section, the resolution of the horizontal mesh at the boundary of the model is 50 mm, but there are multiple regions of higher resolution within this to properly resolve the turbulent wake, see figure below:
[…]

Vertical spacing of the sigma layers in the model, with normalised vertical coordinates (z elevation / depth) and sigma layer numbering from zero (the bed) to the surface (50).

As with comment 8, I would like estimates of the cylinder grid size in wall units (minimum, maximum and mean, for example) to support good boundary layer resolution, or at least to provide quantitative information for reproducing these results with TELEMAC or another CFD code.

- Detailed in the reply above.

10) « Flow depth and discharge were specified at the inlet, with a power law profile specified in the vertical. The flow depth and discharge at the inlet was fixed at 0.6 m and 0.435 m3 s-1, respectively. The outlet was given a fixed discharge of -0.435 m3 s-1, and a free depth to account for the water surface slope »: The way the boundary conditions are set here doesn't make sense if the flow is subcritical. Please explain.
--
Reply: For each iteration the free surface is first solved using the wave equation, then the velocities are solved. The velocity at the boundary will then be scaled to give the prescribed outflow discharge whilst maintaining the cross-sectional shape of the flow field.

I still don't understand this set of boundary conditions. To me, a natural choice would be: an imposed discharge upstream and an imposed flow height upstream. The predicted slope of the free surface would represent the required pressure gradient to sustain the imposed discharge. If bedforms are generated and evolve over time, the friction will increase and so will the slope of the free surface from upstream to downstream. But it should be slow compared with the hydrodynamic time-scale.

- We did exactly that and now clarified in Lines 684-687:

"Flow depth and discharge were specified at the inlet (upstream boundary), with a power law for velocity profile specified in the vertical. The flow depth inlet was fixed at 0.6 m high with a discharge of 0.435 m^3 s^{-1}. The outlet (downstream boundary) was given a fixed discharge of -0.435 m^3 s^{-1}, and a free depth to account for the water surface slope."

- thank you once again for your time and your constructive reviews.

Reviewer #3 (Remarks to the Author):

Unsworth et al. have revised their manuscript to thoroughly address my suggestions and comments, including those on scour protection, decommissioning, and marine spatial planning. In response to other reviewers' suggestions, Unsworth et al. have further strengthened the manuscript by including field data from two wind farms that help verify the real-world application of their model and laboratory experiments. As such, I recommend that the manuscript be considered for acceptance to Nature Communications.
-Avery Paxton

Thank you

Cited papers:

Barton, Alan.J. (2019) 'Blue Kenue enhancements from 2014 to 2019.', *XXVIth TELEMAC-MASCARET User Conference, 15th to 17th October 2019, Toulouse*. Available at: https://doi.org/10.5281/zenodo.3611511.

Biron, P.M. *et al.* (1998) 'Sensitivity of bed shear stress estimated from vertical velocity profiles: the problem of sampling resolution', *Earth Surface Processes and Landforms*, 23(2), pp. 133–139. Available at: https://doi.org/10.1002/(SICI)1096-9837(199802)23:2%253C133::AID-ESP824%253E3.0.CO;2-N.

Biron, P.M. *et al.* (2004) 'Comparing different methods of bed shear stress estimates in simple and complex flow fields', *Earth Surface Processes and Landforms*, 29(11), pp. 1403–1415. Available at: https://doi.org/10.1002/esp.1111.

Bourgoin, A. (2019) *Modélisation de la turbulence engendrée par la morphologie dans le Raz Blanchard : approche régionale avec TELEMAC-LES*. Laboratoire universitaire des sciences appliquées de cherbourg.

Bourgoin, A. *et al.* (2021) 'Use of Large-Eddy Simulation for the bed shear stress estimation over a dune', *International Journal of Sediment Research*, 36(6), pp. 687–695. Available at: https://doi.org/10.1016/j.ijsrc.2019.10.002.

Bourgoin, A.C.L. *et al.* (2020) 'Turbulence characterization at a tidal energy site using large-eddy simulations: case of the Alderney Race', *Philosophical Transactions of the Royal Society A: Mathematical, Physical and Engineering Sciences*, 378(2178), p. 20190499. Available at: https://doi.org/10.1098/rsta.2019.0499.

Dyer, K.R. (1986) *Coastal and Esturine Sediment Dynamics*. Chichester: Wiley.

Escauriaza, C. and Sotiropoulos, F. (2011) 'Initial stages of erosion and bed form development in a turbulent flow around a cylindrical pier', *Journal of Geophysical Research*, 116(F3), p. F03007. Available at: https://doi.org/10.1029/2010JF001749.

Jeon, J. and Kang, S. (2024) 'A Comparative Analysis of Logarithmic Law, Reynolds Stress, and Turbulence Kinetic Energy Methods for Bed Shear Stress Estimation in Complex Open-Channel Flow', *Water Resources Research*, 60(6), p. e2023WR036153. Available at: https://doi.org/10.1029/2023WR036153.

Kim, S.-C. *et al.* (2000) 'Estimating Bottom Stress in Tidal Boundary Layer from Acoustic Doppler Velocimeter Data', *Journal of Hydraulic Engineering*, 126(6), pp. 399–406. Available at: https://doi.org/10.1061/(ASCE)0733-9429(2000)126:6(399).

Manes, C. and Brocchini, M. (2015) 'Local scour around structures and the phenomenology of turbulence', *Journal of Fluid Mechanics*, 779, pp. 309–324. Available at: https://doi.org/10.1017/jfm.2015.389.

Nikuradse, J. (1933) *Stromungsgesetze in rauhem rohren (laws of turbulent pipe flow in smooth pipes)*. VDI-Forschungsheft: Germany.

Pope, N.D., Widdows, J. and Brinsley, M.D. (2006) 'Estimation of bed shear stress using the turbulent kinetic energy approach—A comparison of annular flume and field data', *Continental Shelf Research*, 26(8), pp. 959–970. Available at: https://doi.org/10.1016/j.csr.2006.02.010.

Spalart, P. and Allmaras, S. (1992) 'A one-equation turbulence model for aerodynamic flows', *30th Aerospace Sciences Meeting and Exhibit*. *30th Aerospace Sciences Meeting and*

Exhibit, Reno,NV,U.S.A.: American Institute of Aeronautics and Astronautics. Available at: https://doi.org/10.2514/6.1992-439.

Van den Berg, J. and Van Gelder, A. (1993) ‘A new bedform stability diagram, with emphasis on the transition of ripples to plane bed in flows over fine sand and silt’, *Alluvial Sedimentation (eds M. Marzo and C. Puigdefábregas)*. Available at: https://doi.org/10.1002/9781444303995.ch2.

Van Rijn, L.C. (2007) ‘Unified View of Sediment Transport by Currents and Waves. I: Initiation of Motion, Bed Roughness, and Bed-Load Transport’, *Journal of Hydraulic Engineering*, 133(6), pp. 649–667. Available at: https://doi.org/10.1061/(ASCE)0733-9429(2007)133:6(649).

Williams, J.J., Bell, P.S. and Thorne, P.D. (2003) ‘Field measurements of flow fields and sediment transport above mobile bed forms’, *Journal of Geophysical Research: Oceans*, 108(C4), p. 2002JC001336. Available at: https://doi.org/10.1029/2002JC001336.

Plot directly taken from Manes & Brocchini 2015 – Figure 4. It shows the dimensionless scour depths versus relative coarseness: The data combines several (more recent) studies: (Ettema 1980; Sheppard et al. 2004; Ettema et al. 2006; Lança et al. 2013).

REDACTED

Ettema, Robert, Gokhan Kirkil, and Marian Muste. 2006. "Similitude of Large-Scale Turbulence in Experiments on Local Scour at Cylinders." *Journal of Hydraulic Engineering* 132 (1): 33–40. https://doi.org/10.1061/(ASCE)0733-9429(2006)132:1(33).

Lança, Rui M., Cristina S. Fael, Rodrigo J. Maia, João P. Pêgo, and António H. Cardoso. 2013. "Clear-Water Scour at Comparatively Large Cylindrical Piers." *Journal of Hydraulic Engineering* 139 (11): 1117–25. https://doi.org/10.1061/(ASCE)HY.1943-7900.0000788.

Sheppard, D. Max, Mufeed Odeh, and Tom Glasser. 2004. "Large Scale Clear-Water Local Pier Scour Experiments." *Journal of Hydraulic Engineering* 130 (10): 957–63. https://doi.org/10.1061/(ASCE)0733-9429(2004)130:10(957).
